# A meta-analysis of acetogenic and methanogenic microbiomes in microbial electrosynthesis

**DOI:** 10.1038/s41522-022-00337-5

**Published:** 2022-09-23

**Authors:** Simon Mills, Paolo Dessì, Deepak Pant, Pau Farràs, William T. Sloan, Gavin Collins, Umer Zeeshan Ijaz

**Affiliations:** 1 School of Biological and Chemical Sciences, National University of Ireland Galway, University Road, Galway, H91 TK33 Ireland; 2 Energy Research Centre, Ryan Institute, National University of Ireland Galway, University Road, H91 TK33 Galway, Ireland; 3 Separation and Conversion Technology, Flemish Institute for Technological Research (VITO), Boeretang 200, 2400 Mol, Belgium; 4 Infrastructure and Environment Research Division, School of Engineering, University of Glasgow, Oakfield Avenue, Glasgow, G12 8LT United Kingdom

**Keywords:** Microbial ecology, Bioremediation

## Abstract

A meta-analysis approach was used, to study the microbiomes of biofilms and planktonic communities underpinning microbial electrosynthesis (MES) cells. High-throughput DNA sequencing of 16S rRNA gene amplicons has been increasingly applied to understand MES systems. In this meta-analysis of 22 studies, we find that acetogenic and methanogenic MES cells share 80% of a cathodic core microbiome, and that different inoculum pre-treatments strongly affect community composition. Oxygen scavengers were more abundant in planktonic communities, and several key organisms were associated with operating parameters and good cell performance. We suggest *Desulfovibrio* sp. play a role in initiating early biofilm development and shaping microbial communities by catalysing H_2_ production, to sustain either *Acetobacterium* sp. or *Methanobacterium* sp. Microbial community assembly became more stochastic over time, causing diversification of the biofilm (cathodic) community in acetogenic cells and leading to re-establishment of methanogens, despite inoculum pre-treatments. This suggests that repeated interventions may be required to suppress methanogenesis.

## Introduction

Microbial electrosynthesis (MES) is an emerging electro-bioprocess for CO_2_ conversion to green chemicals, which promises to complement other nascent and established technologies aimed at decarbonizing industrial production^1^. In MES cells, specific microorganisms – electrotrophs – form a biofilm and catalyse CO_2_ reduction by accepting electrons from a solid cathode electrode, either directly, or by mediated pathways^2^. Bioelectrocatalytic CO_2_ reduction offers significant advantages over thermo- and electro-catalytic counterparts, since microorganisms act as inexpensive, self-regenerating catalysts active under mild temperature and pressure conditions, potentially achieving coulombic efficiencies exceeding 90%^3,4^. Furthermore, if mixed-species consortia are used as inoculum, flexible and resilient biofilm communities are formed, which can be adapted to synthesise a wide array of products from flue gas streams containing variable CO_2_ concentrations and various contaminants. However, MES is constrained by the low current density that can be supported by the biological catalyst, which remains one to two orders of magnitude lower than those achieved in state-of-art electrolysers^5^.

MES was first described by Nevin et al.^6^, who reported that the acetogenic microorganism *Sporomusa ovata* is capable of reducing CO_2_ by using electrons accepted from a cathode electrode. Subsequently, this function was proposed for several other microorganisms, including other *Sporomusa* sp. as well as *Clostridium* sp. and *Moorella* sp., and even for archaeal species such as *Methanobacterium* sp. and *Methanococcus* sp.^7^. However, discerning cathodic, direct electron transfer from the common H_2_-mediated CO_2_ reduction pathway is highly challenging. Indeed, our understanding of electron transfer mechanisms and metabolic pathways in MES is further complicated when complex, undefined microbial communities, rather than pure cultures, are used as inoculum. In such cases, the MES product spectrum, yields, production rates, and coulombic efficiency will be strictly linked to the distribution, abundance and networking of the microbial taxa underpinning bioelectrochemical conversion processes. Therefore, understanding and predicting the composition of MES microbial communities that develop from different inoculum sources could help to optimize the process.

Anaerobic and activated sludges are popular inocula for MES cells, and are typically pre-treated to eliminate methanogenic archaea when methane is not the target product^8^. Pretreatment is essential, even when using activated sludge as inoculum, since it will contain acetoclastic and hydrogenotrophic methanogenic species (albeit at concentrations 1–2 orders of magnitude lower than in anaerobic sludge) since the nuclei of the activated sludge flocs are typically anaerobic^9^. Heat shock^8,10^ and chemical pre-treatment with 2-bromoethanesulphonic acid (BESA)^11,12^ are the most commonly applied techniques to inhibit methanogens in MES cells. However, the possible impact of inoculum pre-treatment on other members of the electrosynthetic community has not yet been elucidated.

Microorganisms colonise MES cells either in planktonic form, or by forming a biofilm on the cathode surface, resulting in two different microbial communities^11,13^, of which the various roles and interactions have not been fully elucidated. By studying the metagenome of a mixed-species MES culture producing acetate from CO_2_, Marshall et al.^14^ developed a model in which three dominant microorganisms (i.e., the electrotroph *Desulfovibrio*, the acetogen *Acetobacterium* and the microaerophilic *Sulfurospirillum*) were implicated in the predominant metabolic pathways. Furthermore, Cabau-Painado et al.^15^ developed a model to predict microbial kinetics in MES, and link them to the cell performance. However, defining a general model to link microbial dynamics and productivity of the highly variable MES processes is arduous considering the scale of information currently available. A necessary first step in this direction is to better understand the microbial communities underpinning MES processes, including relevant microbial interactions; evolution in response to operating parameters (primarily pH, temperature, cathode potential); and how they affect production in MES cells.

High-throughput DNA sequencing, especially of 16S rRNA gene amplicons, using platforms such as Illumina Miseq, has provided a powerful tool to study microbial communities in biotechnologies^16^. Such microbiomics have also been increasingly adopted in MES research (Fig. 6). However, only 27.4% of MES-related studies had deposited their sequencing data into public databases. Nonetheless, several terabytes of data are available on online repositories such as Short Read Archive (SRA), from MES cells enriched using various inocula and operated under a variety of conditions. However, single-study results have low reproducibility due to the highly variable experimental set-ups used. Meta-analysis approaches, whereby results from different studies are merged to increase the statistical significance and to offer a global perspective, will help in elucidating the microbial dynamics of MES cells. Hence, in this study, publicly available sequencing data, along with data kindly provided by several authors, were collated to provide a meta-analysis of 16 S rRNA gene sequences from MES cells. The core microbiomes of the biofilm and planktonic communities in acetogenic and methanogenic cells were identified. Furthermore, the effects on microbial communities of common inoculation and operational strategies were assessed, providing valuable information for optimisation of MES cell performance.

## Results and discussion

### Statistical distribution of the samples

The statistical distribution of the samples across the parameters analysed is supplied in the Supplementary Fig. 1. All 261 samples were categorised as either inoculum sources (9%), planktonic (29%) or cathodic (62%) MES communities. The MES cells were mainly inoculated with anaerobic sludge (62%), or less commonly with activated sludge (22%) or other inocula (16%), and were in most cases enriched under H_2_:CO_2_ or in previously-operated MES cells. Inocula without any pre-treatment were used in 33% of samples, mainly in methanogenic MES cells, whereas pre-treatment using either 2-bromoethanesulfonic acid (BESA) (51%) or heat shock (16%) was applied to biomass used in most of the acetogenic MES cells.

Samples originated from MES cells operated at a range of temperatures: below 30 °C (36%), between 30 and 45 °C (52%), and above 45 °C (12%). Sampling timepoint (i.e. the number of operating days elapsed at the moment of sampling) was below 50 d (28%), between 50 and 100 d (17%), between 100 and 200 d (21%), and above 200 d (34%). The pH ranged between 5 and 8 in 69% of cases, and was <5 or >8 in 24% and 7% of cases, respectively. The cathodic potential applied, ranged from −0.7 to −1.1 V *vs* Ag/AgCl, but mostly at either −0.8 V (26%) or −1.0 V (44%). Only one study^17^ was performed in galvanostatic mode at an applied current of 10 A/m^2^, whereas one study^18^ controlled the cell voltage with a power supply. Acetate (68%) or methane (29%) were the main products of the MES cells included in this meta-analysis, with widely variable production rates and coulombic efficiencies. Low concentrations of other products such as ethanol, butanol, butyrate, propionate and caproate were seldomly reported.

### Key differences between cathodic and planktonic communities in acetogenic and methanogenic MES cells

**Fig. 1:**
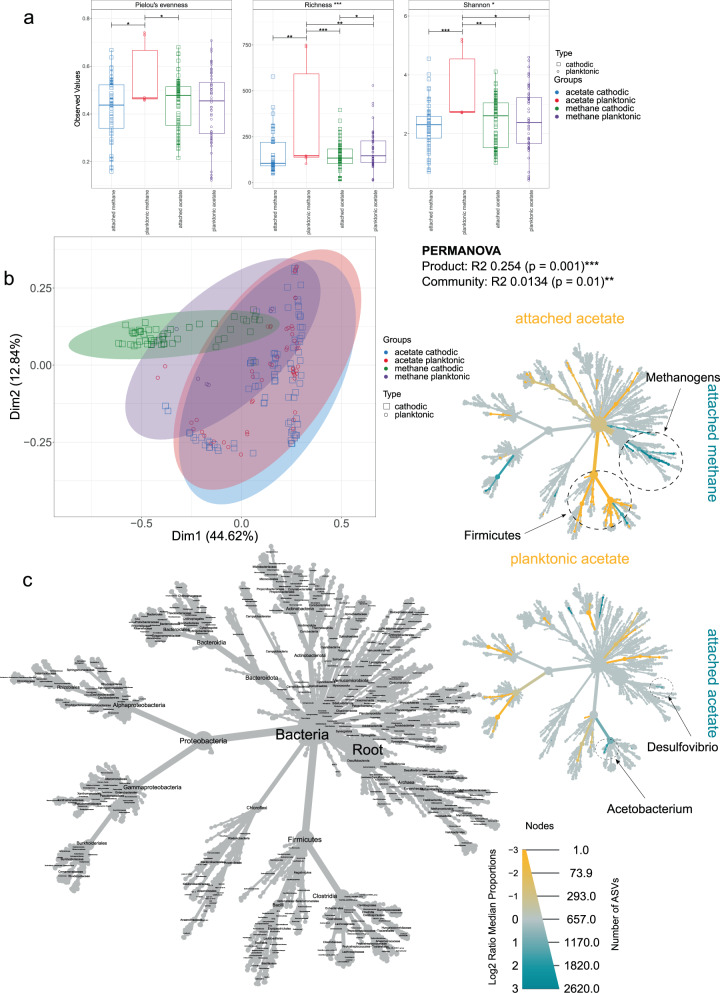
Comparison of cathodic and planktonic microbial communities. **a** Alpha Diversity Indices; Pielou’s Evenness, Rarefied Richness and Shannon Entropy for cathodic and planktonic communities in acetogenic and methanogenic cells. In the boxplots, center value lines indicate the median, boxes indicate the lower/upper quartiles (25%/75%) and lines extending parallel from the boxes (whiskers) show the variability outside the upper and lower quartiles. Lines of significance depict significant differences as follows: * (*p* < 0.05), ** (*p* < 0.01), or *** (*p* < 0.001) based on ANOVA. **b** Principal Component Analysis (Weighted Unifrac) of cathodic and planktonic communities in acetogenic and methanogenic cells, where ellipses were drawn using 95% confidence intervals based on standard deviation. **c** Heat Trees depicting differential abundances of taxa among the groups in question. The circle size and the colour intensity reflect the species abundance and the log2 median proportion between the two groups respectively.

Samples were grouped by product (acetate or methane) and community type (planktonic or cathodic) for alpha diversity analysis, which indicated that species richness in the acetogenic planktonic community was only slightly higher than in the cathodic community but the difference was still deemed to be statistically significant (Fig. 1). The methanogenic planktonic community was significantly richer and more diverse than the cathodic community. However, the methanogenic planktonic group included only six samples and, therefore, this result is not conclusive. Cathodic and planktonic acetogenic communities clustered together in PCoA and were similar in both composition and phylogeny (Fig. 1). Samples from methanogenic MES systems clustered separately from the acetogenic MES samples in PCoA indicating that their microbial community compositions differed (Fig. 1). Indeed, PERMANOVA indicated that the main electrosynthesis product (acetate or methane) accounted for approximately 25% (*p* < 0.001) of the variance across all samples.

*Acetobacterium* sp. were the most relatively abundant genus in the cathodic community (10–70% relative abundance; Supplementary Fig. 2). Exceptions included the two studies reporting thermophilic MES of acetate^19,20^, in which *Moorella* and *Caloribacterium* (at 50 °C), and *Tepidiphilus* and *Coprothermobacter* (at 70 °C), were enriched. Furthermore, Alqahtani et al.^21^ enriched a distinct community dominated by Proteobacteria, such as *Marinobacter*, and Firmicutes, such as *Halanaerobiaceae*, in MES cells inoculated with marine sediment using either a synthetic medium (10% salinity) or real brine (25% salinity) as electrolyte. Since the communities from these thermophilic and halophilic studies were so different from the other (mesophilic or ambient) studies, and only represented 31 samples, these have been excluded from all analysis other than the taxa plots (Supplementary Fig. 2). *Methanobacterium* sp. were dominant in methanogenic MES, with relative abundances often exceeding 50%. The exception was from Alqahtani et al.^22^, who enriched a distinct community with abundant *Methanococcus* in saline MES reactors (3.5% salinity) inoculated with salt marsh and mangrove sediments. Both *Methanobacterium* and *Methanococcus* are hydrogenotrophic archaea, which are generally recognised as the prevalent methane-producers in bioelectrochemical systems^23^.

We identified significant differences between the acetogenic and methanogenic cathodic communities using differential heat trees (Fig. 1). In particular, Firmicutes and Archaea (Fig. 1), which were more prominent in acetogenic and methanogenic systems respectively. The main difference between cathodic and planktonic acetogenic communities was that *Acetobacterium* sp. and *Desulfovibrio* sp. were significantly more abundant in the cathodic community (Fig. 1). Both genera were previously identified as key players in MES. *Desulfovibrio* sp. were hypothesised to accept electrons from the cathode through cytochromes, hydrogenases and/or formate dehydrogenase, and to subsequently reduce protons to molecular H_2_, and/or reduce CO_2_ to formate^14^. H_2_ and formate are then converted to acetate by *Acetobacterium* sp. through the Wood-Ljungdahl pathway^14^. Two more microbial genera (*Arcobacteriaceae* sp. and an uncultured *Actinobacteria*) were found upregulated within the cathodic community. It was suggested that certain *Actinobacteria* sp. can uptake electrons from the cathode^24^, whereas *Arcobacter* sp. is a sulphate reducer capable of CO_2_ fixation under autotrophic conditions, widely reported as member of the MES cathodic community^25,26^. Several microorganisms were more abundant in the planktonic community of acetogenic cells, including *Gammaproteobacteria* (including the aerobes *Stenotrophomonas* sp. and *Pseudomonas* sp.), *Bacilli*, *Actinobacteria* and *Bacteroidia* (Fig. 1). The growth of aerobic microorganisms is likely due to oxygen diffusion from the anodic compartment towards the cathodic compartment through the ion exchange membranes^27^.

### Core microbiome of acetogenic and methanogenic cells

**Fig. 2:**
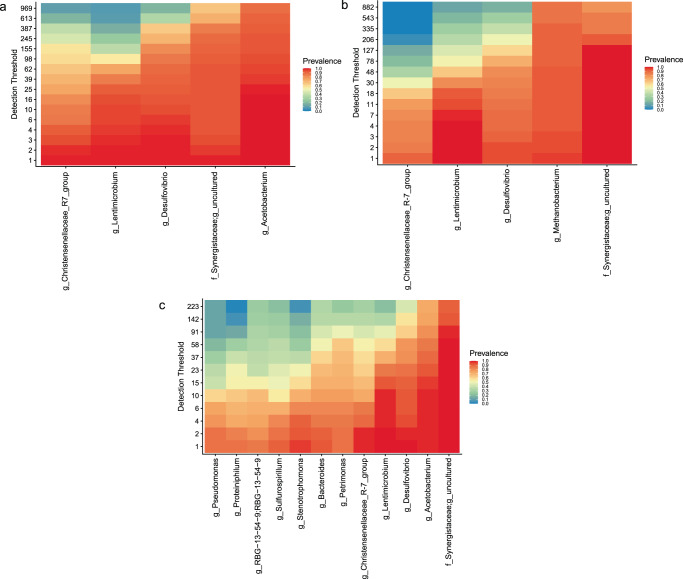
Core microbiomes of acetogenic and methanogenic MES cells. **a** Highly prevalent taxa in the core microbiomes of (**a**) acetogenic cathodic, **b** methanogenic cathodic and **c** acetogenic planktonic communities. Detection thresholds (number of reads) are shown on the y-axis.

Defining the core microbiome as including genera present in 85% of the samples considered, we identified five qualifying genera from the acetogenic and methanogenic cathodic communities, and twelve genera in the acetogenic planktonic community, which were plotted at different detection thresholds (numbers of reads) (Fig. 2). Given the few samples available, the core methanogenic planktonic community was not considered. Interestingly, the core microbiome of the acetogenic and methanogenic cathodic community was very similar and differed by only one genus. Four genera (*Christensenellaceae*_R − 7_group; *Desulfovibrio* sp.; *Lentimicrobium* sp.; and an uncultured genus in the *Synergistaceae* family) were present in the cathodic core microbiomes of both acetogenic and methanogenic MES cells. *Acetobacterium* sp. were additionally present in acetogenic samples and *Methanobacterium* sp. were present in methanogenic samples (Fig. 2). Therefore, four shared genera may be keystone members in MES. The analysis confirms the key role of *Desulfovibrio* sp., which is believed to be the main hydrogen-producer in MES, providing reducing equivalents to autotrophic microorganisms, such as *Acetobacterium* sp. or *Methanobacterium* sp. The role of the other core genera remains unclear, however, it may be the case that they have an unknown syntrophic association with either *Acetobacterium* sp or *Methanobacterium* sp. *Synergistaceae* were present in the core microbiome at thresholds up to 882 reads in the cathodic community (Fig. 2), likely acting as syntrophic partners of either acetogenic or methanogenic populations. In fact, the *Synergistaceae* were previously described, along with *Christensenellaceae*, as part of the “highly abundant core community” of a full-scale anaerobic digestion plant^28^, suggesting their importance in multiple anaerobic bioprocesses.

The five members of the acetogenic cathodic core microbiome were also part of the planktonic core community (Fig. 2). However, the planktonic core community was more diverse, including seven more genera. The presence of aerobic microorganisms, such as *Stenotrophomonas* sp. and *Pseudomonas* sp., in the planktonic core microbiome suggests that oxygen intrusion occurred in most MES studies, highlighting the importance of oxygen scavengers in maintaining anaerobic conditions near the cathode.

### Impact of inoculum source, and pre-treatment, on community development

**Fig. 3:**
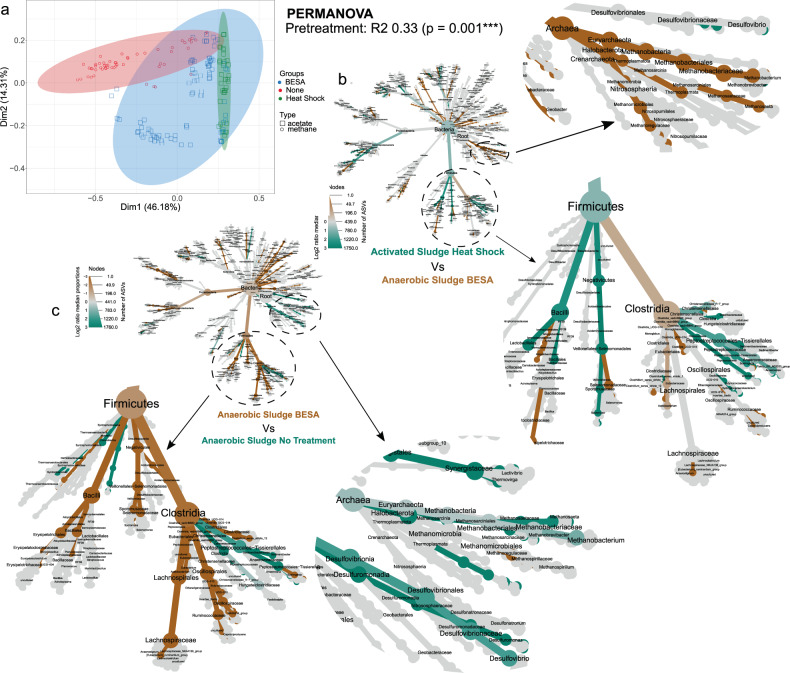
Effect on inoculum pre-treatment on the microbial communities in MES cells. **a** Principal Component Analysis (Weighted Unifrac) showing acetogenic and methanogenic samples clustered by pretreatment method where ellipses were drawn using 95% confidence intervals based on standard deviation. **b** Differential heat tree depicting differently abundant taxa between two groups based on seeding strategy (heat shocked activated sludge Vs BESA treated anaerobic sludge). The circle size and the colour intensity reflect the species abundance and the log2 median proportion between the two groups respectively. **c** Differential heat tree depicting differently abundant taxa between two groups based on seeding strategy (untreated anaerobic sludge Vs BESA treated anaerobic sludge). The circle size and the colour intensity reflect the species abundance and the log2 median proportion between the two groups respectively. Full size, high-resolution heat trees are provided in the Supplementary Information (Supplementary Figs. 6, 7).

The majority of cells were inoculated with either anaerobic or activated sludge (Supplementary Fig. 1), which had variable microbial community composition (Supplementary Fig. 3). For acetogenic MES, inocula were commonly pre-treated with BESA or by heat-shock. Significantly higher richness and evenness was observed in communities originating from heat-shock-pretreated inocula than in BESA-pretreated inocula (Supplementary Fig. 4). Pre-treated samples (BESA or heat shock) clustered together in PCoA (Fig. 3), and pre-treatment explained 33% of the total variability among samples (*p* = 0.001). Clustering based on pretreatment (Fig. 3) also aligned closely to clustering based on the product (acetate or methane), indicating that pre-treatment was an important factor influencing the MES microbiome and the metabolites produced.

Anaerobic sludge pre-treated with BESA and activated sludge pre-treated by heat-shock were the most common inocula used in acetogenic MES cells. Firmicutes were more abundant in heat-treated, activated sludge, including spore-forming microorganisms such as *Lactobacillales*, *Sporomusaceae* and several *Clostridia* (Fig. 3). Full-size, high-resolution heat trees are provided in the Supplementary Information (Supplementary Figs. 6, 7). The ability of these microorganisms to form spores^29^ infers an advantage that selects them in heat-shock pretreated inocula. Undesired methanogens, such as *Methanobacterium* sp. and *Methanosaeta* sp., were more abundant in BESA-pretreated anaerobic sludge (Fig. 3), which suggests that heat shock is a better pretreatment to supress methanogenesis. However, heat shock is less specific and can also affect other important organisms such as *Acetobacterium* sp., which were significantly less abundant in MES cells seeded with heat-shocked activated sludge (Fig. 3). Microorganisms involved in chain elongation and propionate production, such as *Caproiciproducens* sp. and *Propionibacterium* sp., were also less abundant when heat-shocked activated sludge was used as inoculum. Hence, heat shock should be avoided when targeting middle-chain fatty acids, and more specific strategies to inhibit methanogenic activity should then be considered.

**Fig. 4:**
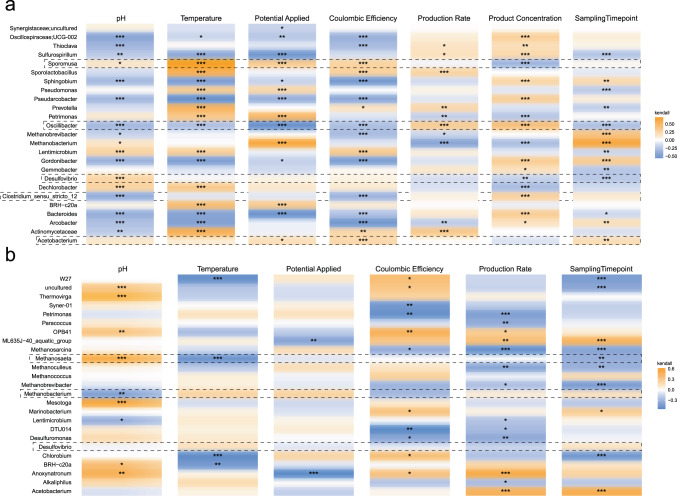
Correlation between taxa and environmental parameters. Correlation heatmap of the 25 most abundant genera from (**a**) acetogenic, and (**b**) methanogenic cells with environmental variables. Kendall correlations between the taxa and the environmental variables were calculated. Significance levels are indicated by asterisks as *(*p* < 0.05), **(*p* < 0.01) or ***(*p* < 0.001).

As expected, methanogenic archaea were less abundant in (typically acetogenic) MES cells seeded with BESA pre-treated anaerobic sludge than in (typically methanogenic) MES cells seeded with untreated sludge (Fig. 3). However, methanogens such as *Methanobrevibacter* sp. and *Methanobacterium* sp. correlated positively with the sampling timepoint in acetogenic MES cells (Fig. 4), suggesting that methanogens are only temporarily inhibited by BESA pretreatment. This could be due to either adaptation of the community, or degradation of the inhibitor. It was indeed reported that sulphate-reducing bacteria, including *Desulfovibrio* sp., can reduce BESA to H_2_S^12^. Therefore, pre-treatment needs to be periodically repeated to reliably inhibit methanogenesis. However, in this study, *Desulfovibrio* sp. was lower in abundance in samples treated with BESA, which disagrees with its possible role in BESA reduction in MES cells. Conversely, several acetogenic Firmicutes (including *Acetobacterium* sp. and *Sporomusa* sp.), and potential chain-elongating microorganisms (*Caproiciproducens* sp. and *Clostridium_sensu_stricto_12*), were more abundant in BESA-treated MES cells, potentially favoured when competition from methanogens is removed. Once again, this result indicates that BESA may offer a better pre-treatment when targeting the production of middle-chain fatty acids.

### Response of microbial communities to MES operating conditions

Several significant correlations were identified between genera in acetogenic, and methanogenic, MES cells and operating, or performance, parameters (pH, temperature, potential applied, coulombic efficiency, production rate and product concentration) (Fig. 4). *Sporomusa* sp. correlated positively with temperature (in the range 10–35 °C, since thermophilic samples were excluded) in acetogenic cells and were more abundant under mesophilic conditions. However, *Sporomusa* sp. have also been observed to grow autotrophically in MES cells at lower temperatures^30^. Other key genera, such as *Clostridium* sp., *Acetobacterium* sp. and *Desulfovibrio* sp., did not correlate with temperature. *Desulfovibrio* sp. did, however, correlate positively with pH. This could explain the higher abundance of *Desulfovibrio* in the cathodic rather than planktonic community, as the local pH at the surface of the cathode is higher due to alkalization phenomena^31^. *Clostridium* sp. and *Oscillibacter* sp. correlated negatively with pH, indicating that they prefer mildly acidic conditions. Interestingly, *Sporomusa* sp. correlated negatively with acetate concentration in the catholyte, suggesting lower tolerance for product accumulation than other acetogens, such as *Acetobacterium* sp. (Fig. 4).

Significant correlations were also found between several organisms and the cathodic potential applied to the MES cell (ranging from −0.8 to −1.1 V *vs* Ag/AgCl) (Fig. 4). *Sporomusa* sp. correlated positively with potential, suggesting its ability to perform electrosynthesis at relatively higher potentials than other acetogenic microorganisms. Acetogenesis with *Sporomusa ovata* was observed at potentials as high as −0.4 *vs* SHE (−0.6 *vs* Ag/AgCl)^6^, although the electron transfer mechanism for CO_2_ reduction at such potential is still debated. While direct electron transfer from the cathode to microorganisms appeared as the most reasonable hypothesis, due to the insufficient potential to generate hydrogen abiotically, it was recently argued that microbially-secreted metabolites/cell-components might enable H_2_ evolution at higher potentials^32^. This is particularly true in mixed-culture MES, wherein several microorganisms can participate in H_2_ catalysis at the cathode, thus making H_2_-mediated electron transfer the prevalent acetogenic route. Furthermore, acetogenic microorganisms can increase the onset potential and evolution rate of H_2_ by promptly consuming it and maintaining low partial pressure near the cathode^33^.

The abundance of key microorganisms, such *Desulfovibrio* sp. and *Acetobacterium* sp., did not correlate with the applied potential, suggesting their flexibility, whereas *Sulfurospirillum* sp. preferred low potentials (Fig. 4). *Sulfurospirillum* sp. are capable of carbon fixation using H_2_ as reducing agent, and were suggested to protect the strictly anaerobic members of the community by consuming traces of oxygen often present in MES cathodes, albeit at the cost of acetate consumption^14^. Thus, *Sulfurospirillum* sp. are favoured at low potentials both directly, due to the faster H_2_ evolution, and indirectly, due to the fast accumulation of acetic acid resulting from the abundance of reducing equivalents. Similarly, the preference of *Oscillibacter* sp., suspected chain-elongating microorganisms^11^, for low potential can be attributed to the observed higher acetate production rates. This hypothesis is supported by the fact that both *Sulfurospirillum* and *Oscillibacter* sp. correlated positively with acetate concentration in the catholyte (Fig. 4).

In methanogenic MES cells, several microorganisms, including acetoclastic *Methanosaeta* sp., correlated positively with pH (ranging between 6.8 and 8.5 among the samples). This was not the case for *Methanobacterium* sp., which are often dominant in methanogenic MES under both neutral and slightly alkaline pH conditions. Overall, the methanogenic community appears to be less associated with temperature and applied potential than the acetogenic community (Fig. 4).

### Cathodic microbial community assembly

**Fig. 5:**
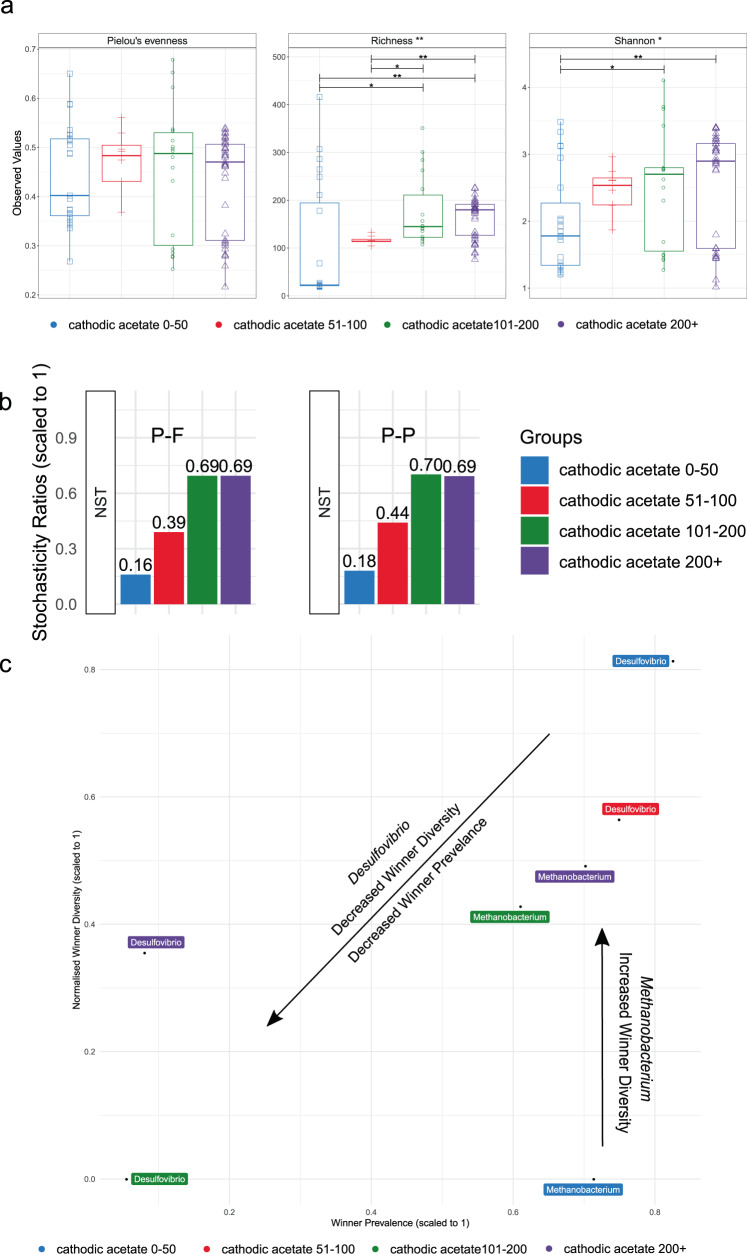
Community assembly processes in cathodic acetogenic communities. **a** Alpha Diversity Indices; Pielou’s Evenness, Rarefied Richness and Shannon Entropy for cathodic acetogenic communities. In the boxplots, center value lines indicate the median, boxes indicate the lower/upper quartiles (25%/75%) and lines extending parallel from the boxes (whiskers) show the variability outside the upper and lower quartiles. Lines of significance depict significant differences as follows: * (*p* < 0.05), ** (*p* < 0.01), or *** (*p* < 0.001) based on ANOVA. **b** Normalized stochasticity ratio (NST) using Ružička metric and Taxa-Richness constraints of proportional-fixed (P-F) and proportional-proportional (P-P) which stipulates that the probabilities of taxa occurrence are proportional to the observed occurrence frequencies, and taxon richness in each sample is either fixed or proportional. **c** Scatter plot indicating winner diversity and winner prevelance for genera exhibiting lottery model-like behaviour (a genera member makes up >90% of the genera’s abundance). Samples in each panel are grouped into four time periods 0–50 d, 51–100 d, 101–200 d and 200 + d.

To assess microbial community assembly processes in the cathodic community over time, samples were grouped into four ranges based on the sampling timepoint (i.e. on the duration of cell operation). Those were*:* 0–50 days, 51–100 days, 101–200 days and 200+ days. Only acetogenic MES samples were considered due to the lack of methanogenic MES samples older than 100 d. Shannon diversity and species richness increased significantly over time from young (<100 d) to old (>100 d) biofilms (Fig. 5). This is an interesting finding, since MES cells are typically operated as semi-closed systems, allowing the entry only of gas, thus excluding immigration as a source of *de novo* richness and diversity.

The study of patterns of microbial community assembly has gained popularity in recent years and may be useful in understanding, and controlling, microbial communities in engineered systems, such as MES cells. This facet of microbial ecology describes the forces governing community development to determine mechanisms and factors controlling microbial diversity^34^. Community assembly mechanisms broadly fit two categories: deterministic community assembly, which indicates that interspecies interactions, or environmental gradients, create niches, influencing patterns of diversity^35,36^; and stochastic community assembly, which implies that community assembly is governed by more-neutral processes, such as ecological drift, birth and death dynamics, speciation, and immigration^35,36^.

NST analysis was applied to consider whether deterministic or stochastic mechanisms dominated cathodic community assembly. A value of >0.50 indicates a community is more influenced by stochastic factors, whereas a value <0.50 indicates deterministic forces are prominent. Early biofilm development was more influenced by deterministic than stochastic processes (Fig. 5). This was likely caused by adaptation of the community to the new environment (i.e., MES cathode and electrolyte) or effects of BESA or heat-shock pre-treatments. In older samples (>100 d), stochasticity in cathode community increased significantly, leading to higher richness and diversity (Fig. 5). Since the relative abundance of acetogen competitors, such as *Methanobacterium* sp., increased over time (Fig. 4), the stochasticity observed in older biofilms may be detrimental to cell performance. Therefore, a management strategy may be required to reduce stochasticity and maintain acetogenic populations. This may include periodic inhibition of the methanogenic population coupled with acetate extraction to limit the toxic effect of product accumulation on the acetogenic community.

Lottery model analysis identified *Methanobacterium* sp. as ‘lottery winners’, indicating that in the *Methanobacterium* genus a single ASV often made up 90% of its abundance. In early biofilm samples (0–50 d) *Methanobacterium* sp. had high winner prevalence but very low winner diversity, meaning that in most (>70%) samples a single *Methanobacterium* ASV was dominant. However, in older biofilms (100 + d) *Methanobacterium* lottery winners were more diverse. Since it is unlikely that new *Methanobacterium* ASVs were introduced by immigration, stochasticity may have caused them to proliferate through acclimatisation, adaptation or even speciation, providing more potential winning ASVs.

The second lottery winner was *Desulfovibrio* sp., which had high winner prevalence and high winner diversity in the young biofilms, strongly suggesting that several members of this genus can quickly colonise the cathode. Winner prevalence and diversity lessened over time, indicating that fewer, though highly competitive, *Desulfovibrio* ASVs dominanted older biofilms. Furthermore, winner behaviour was apparent in fewer samples in aged biofilms, indicating *Desulfovibrio* was gradually outcompeted, with only a few highly competitive ASVs remaining. This result is in agreement with the negative correlation found between *Desulfovibrio* and sampling timepoint (Fig. 4). It is interesting that only two genera were identified as displaying lottery winner behaviour, as this indicates that none of the other genera were dominated by single ASVs. This result suggests that MES cells strongly select for specific *Desulfovibrio* and *Methanobacterium* ASVs, whereas the composition of other genera was more random and varied between studies.

### Correlation between key microorganisms and MES cell performance

*Sporomusa sp* abundance correlated strongly with coulombic efficiency (CE) (Fig. 4). The *Sporomusa* genus includes several highly efficient species, such as *S. ovata* and *S. malonica*, capable of acetogenesis in MES with >90% CE^30^. Such high CE can be attributed to the direct electron uptake capability^37^ and/or to the efficient metabolism of *Sporomusa* sp. using H_2_ as electron donor^30^. *Acetobacterium* sp. abundance also correlated positively with CE, whereas neither *Acetobacterium* nor *Sporomusa* sp. correlated with production rates. Production rates are likely more dependent on the operating parameters (e.g. the concentration of reducing equivalents) than the organism performing acetogenesis. No correlation was found between *Sulfurospirillum* sp. abundance and CE. However, it correlated positively with production rate, confirming the importance of its oxygen scavenging role in MES. As expected, methanogens such as *Methanobacterium* and *Methanobrevibacter* sp. were associated with low CE and/or low acetate production rates. More surprisingly, *Clostridium sensu stricto* 12 correlated negatively with CE, suggesting its role in chain elongation pathways, rather than acetogenesis, as previously hypothesised^38^.

In methanogenic MES cells, neither CE nor methane production rates correlated with *Methanobacterium* sp., which dominates the microbial community of methanogenic MES cells regardless of their productivity. In fact, *Methanobacterium* sp. constituted around 40% of the community of the granular sludge-based electromethanogenic reactor that achieved the highest methane production rate so far, with 85% CE^18^, but was also highly abundant in poorly performing cells (Supplementary Fig. 2). *Methanobacterium* is a highly resilient genus of hydrogenotrophic microorganisms, which prevails in methanogenesis under stressed conditions^28^. In MES cells, such conditions are promoted by local pH gradients at the electrodes; ion migration; and oxygen intrusion from the anode chamber. Interestingly, *Methanosarcina*, sp. which are capable of both hydrogenotrophic and acetoclastic metabolism, were associated with low CE and methane production rates (Fig. 3). Cytochrome-containing archaea, such as some *Methanosarcina* sp., show restricted growth on H_2_ and CO_2_, as well as at least a ten-fold higher H_2_ partial pressure threshold than archaea lacking cytochromes, such as *Methanobacterium*^39^. Thus, it is plausible that in well-performing cells, *Methanobacterium* keeps the H_2_ partial pressure at the cathode low and thus outcompetes *Methanosarcina*, which is abundant in poorly-performing cells due to H_2_ accumulation.

To conclude, meta-analysis revealed the key role of H_2_-producing *Desulfovibrio* electrotrophs in early-stage MES biofilms, suggesting that both acetogenesis and methanogenesis are H_2_-mediated. Heat shock is a more effective pre-treatment than BESA to prevent the onset of methanogenesis, but may hamper product diversification, and should be avoided when targeting middle chain carboxylic acid production. Oxygen-scavenging microorganisms were important members of the core planktonic MES community. Their positive correlation with CE confirmed their important role in efficient MES cells, and that oxygen intrusion from the anode chamber is an unresolved challenge. Richness, diversity and stochasticity in the acetogenic cathodic community increase over time, mainly due to increasing competition from methanogens, suggesting that pre-treatment of the microbial community should be periodically repeated.

## Methods

### Data collection

**Fig. 6:**
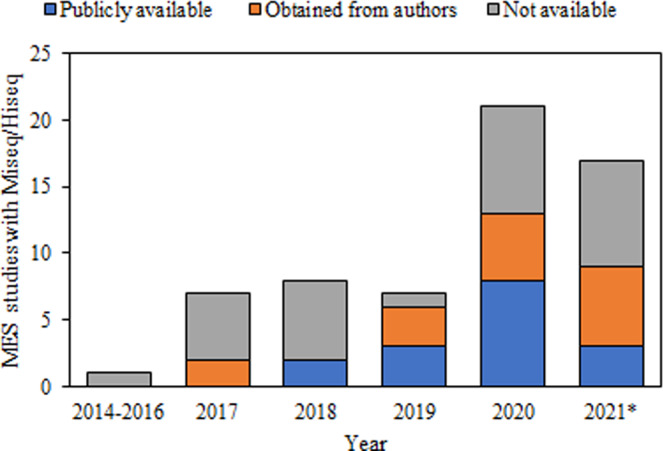
Yearly distribution of articles on MES cells featuring microbial community analysis *via* high-throughput sequencing (Miseq/Hiseq) with universal primers. Suitable publicly available data, along with data requested and obtained from the Authors, was included to the meta-data table. Data from 2021 includes only studies published by June.

Research articles on MES were retrieved using the Google Scholar, Scopus and Elsevier search engines using the keywords “microbial electrosynthesis”, “MES”, “biocathode”, and “bioelectro”. The resulting articles were screened to select the studies that included 16 S rRNA gene sequence analysis on the Illumina Miseq or Hiseq platforms, and which used universal bacterial and archaeal oligonucelotide primers. The reason for curtailing the studies to just those that utilised Illumina platform is based on authors’ recent work where it was shown, based on mock communities, that Illumina platforms are more quantitative with less systematic biases^40^. After initial screening, 61 articles were considered suitable for the study, although sequencing data were available in public databases from only 16 (26.2%) of the studies. Sequencing data from an additional 16 studies were requested, and obtained, from the respective authors, while the remaining 29 articles (46.8%) were regrettably excluded from the study due to the unavailability of the data (Fig. 6).

Ten more articles were excluded in a second screening due to poor sequencing quality and/or unavailability of metadata, resulting in a final total of 22 studies in the meta-analysis. The studies included are listed in the Supplementary Table 1. Publicly available data were downloaded using the Sequence Read Archive (SRA) toolkit provided by the NCBI. Metadata were collected from the NCBI and directly from the respective published articles. A total of 261 samples were included in a meta-data table and categorised based on parameters, including: primers used; region of the 16 S rRNA gene targeted; inoculum and pre-treatment used; microbial community type (cathodic, planktonic or inoculum); sampling timepoint (i.e. in the MES trial); potential/current applied; operating parameters (e.g., pH, temperature); product spectrum, concentration and production rates; and coulombic efficiency. When the required data were not explicitly provided in the text, it was obtained by extrapolating an approximate value from the figures in the manuscript, where possible. When necessary, units of measurement were converted to allow inter-study comparisons.

### Sequence processing

Sequences from each study were processed individually using Qiime2. Since multiple V regions of the 16 S rRNA gene were considered in this study, the DEBLUR algorithm was used to generate amplicon sequence variants (ASVs). DEBLUR was used over DADA2 because, for longer amplicon regions (e.g, V3-4), sometimes DADA2 is unable to give the optimum number of ASVs as the denoising is done separately for forward and reverse reads before finding sufficient overlap to assemble them together. Taxonomy was assigned using a Bayesian Least Common Ancestor (BLCA) approach (using the SILVA138 database) as recommended by Keating et al.^41^, to obtain more sequence-level resolution. Since multiple V-regions were used, and some do not overlap, the idea was to collate together as many ASVs as possible to facilitate the generation of phylogentic tree based on full 16 S rRNA sequences. ASVs from each study (matched to the reference database) were collated in a single biom file and sequences without sequence-level assignments were removed. Our collation strategy is already evaluated to cause minimal loss in beta diversity and is mentioned in detail in Thom et al.^42^, with the code already released as part of Keating et al.^41^. Our analysis suggested that the beta diversity patterns between samples (both visual and statistical cues) were preserved when using a minimal set of well-resolved ASVs at the sequence level, as justified in Figures Supplementary Figs. 8–15. Finally, a phylogenetic tree and a biom file with taxonomy were generated using MAFFT and QIIME2, respectively.

### Statistical analysis

All further statistical analysis was done in R (version 3.6.2) using the collated biom file, taxonomy file and phylogenetic tree. Samples were grouped based on relevant metadata parameters, such as ‘community type’ or the main product synthesised by the MES cell. Alpha and beta diversity analyses were done with the Vegan package. Alpha diversity indices used included: rarefied richness, Shannon entropy and Pielou’s evenness. Beta diversity was observed using Principal Coordinate Analysis (PCoA) with the Weighted Unifrac distance metric. Analysis of variance in relation to explanatory variables in the metadata was done using Vegan’s Adonis() function with the Weighted Unifrac distance metric. This function, referred to as permutational analysis of variance (PERMANOVA), fits linear models to distance matrices and used a permutation test with pseudo-F ratios. The core microbiome was determined by identifying taxa with a minimum prevalence of 85% across all samples considered. Whilst there is no single threshold to describe what is core microbiome, particularly when there can be inter-study variations due to biases accumulated by study design, choice of V region, and extraction protocol, we have followed the authors recommendations in terms of absolute read counts^43^. Correlation analysis was used to identify potential relationships between abundant genera and selected environmental variables using Kendall correlation, a nonlinear measure that is more robust. The p-values for correlations were adjusted for multiple testing using the Benjamini and Hochberg procedure to give significant associations. Taxa with significantly differential abundance between groups were visualised using heat trees with a Wilcoxon p-value test adjusted for multiple comparisons on the proportional microbiome data, as recommended by Foster et al.^44^.

Stochasticity in community assembly was assessed using a Normalized Stochasticity Ratio (NST) with various distance measures (incidence-based: Jaccard, Kulczynski and abundance-based: Ruzcika, Kulczynski) using the NST package. A 50% stochasticity ratio was used to identify more deterministic (<50%) and more stochastic (>50%) assembly^45^. For the calculations, taxa occurrence frequency and the sample richness were constrained as proportional (P) or fixed (F) in the combinations PF or PP, and 1,000 randomizations were performed for each model. We have adopted this approach after original authors’ detailed benchmarking of different choices available. Permutational multivariate ANOVA (PANOVA) was then used to assess statistical significance.

A competitive lottery model was used to identify ASVs that dominated their clade (i.e. genera)^46,47^. The model assumes competition among phylogenetically similar species, which will occasionally result in one species, or ASV, dominating by making up >90% of the abundance within that clade (based on a stickbreaking model used on simulated communities)^46^. Here, the lottery ‘winners’ were identified as ASVs dominating a particular genus. Genera with lottery-type behaviour (i.e. containing lottery winners) were then plotted in terms of winner prevalence (proportion of samples having a ‘winner ASV’ among that genus) and winner diversity (the diversity of winning ASVs in samples from which winners were observed). Diversity values closer to 0 suggest that a single ASV consistently dominates that genus across all samples, whilst high diversity values suggest more evenly spread ASVs as winners in that group.

## Supplementary information

Supplementary Material

## Data Availability

E- supplementary data of this work can be found in online version of the paper. A list of all studies used in this meta-analysis has been provided in the supplemental material. Publically available sequencing data has been obtained from the NCBI sequence Read Archive. Data from studies which are not publically available were provided upon request by the authors.

## Code availability

All code used in this manuscript has been made available at https://userweb.eng.gla.ac.uk/umer.ijaz/#bioinformatics

## References

[1] Grim RG (2020). Transforming the carbon economy: challenges and opportunities in the convergence of low-cost electricity and reductive CO_2_ utilization. Energy Environ. Sci..

[2] Karthikeyan R, Singh R, Bose A (2019). Microbial electron uptake in microbial electrosynthesis: a mini-review. J. Ind. Microbiol. Biotechnol..

[3] Dessì P (2021). Microbial electrosynthesis: Towards sustainable biorefineries for production of green chemicals from CO_2_ emissions. Biotechnol. Adv.

[4] Jourdin L, Freguia S, Flexer V, Keller J (2016). Bringing high-rate, CO_2_‑based microbial electrosynthesis closer to practical implementation through improved electrode design and operating conditions. Environ. Sci. Technol..

[5] Claassens NJ, Cotton CAR, Kopljar D, Bar-Even A (2019). Making quantitative sense of electromicrobial production. Nat. Catal..

[6] Nevin KP, Woodard TL, Franks AE, Summers ZM, Lovley DR (2010). Microbial Electrosynthesis: Feeding Microbes Electricity To Convert Carbon Dioxide and Water to Multicarbon Extracellular Organic Compounds. MBio.

[7] Logan BE, Rossi R, Ragab A, Saikaly PE (2019). Electroactive microorganisms in bioelectrochemical systems. Nat. Rev. Microbiol..

[8] Izadi P, Fontmorin JM, Godain A, Yu EH, Head IM (2020). Parameters influencing the development of highly conductive and efficient biofilm during microbial electrosynthesis: the importance of applied potential and inorganic carbon source. npj Biofilms Microbiomes.

[9] Wu W (1987). Cultivation of anaerobic granular sludge in UASB reactors with aerobic activated sludge as seed. Water Res..

[10] Izadi P, Fontmorin JM, Virdis B, Head IM, Yu EH (2021). The effect of the polarised cathode, formate and ethanol on chain elongation of acetate in microbial electrosynthesis. Appl. Energy.

[11] Dessì P (2021). Carboxylic acids production and electrosynthetic microbial community evolution under different CO_2_ feeding regimens. Bioelectrochemistry.

[12] Bian B, Xu J, Katuri KP, Saikaly PE (2021). Resistance assessment of microbial electrosynthesis for biochemical production to changes in delivery methods and CO_2_ flow rates. Bioresour. Technol..

[13] Song YE (2022). Biofilm matrix and artificial mediator for efficient electron transport in CO_2_ microbial electrosynthesis. Chem. Eng. J..

[14] Marshall CW (2017). Metabolic Reconstruction and Modeling Microbial Electrosynthesis. Sci. Rep..

[15] Cabau-Peinado O, Straathof AJJ, Jourdin L (2021). A General Model for Biofilm-Driven Microbial Electrosynthesis of Carboxylates From CO_2_. Front. Microbiol..

[16] Thompson LR (2017). A communal catalogue reveals Earth’s multiscale microbial diversity. Nature.

[17] Chu N (2020). Waste C1 Gases as Alternatives to Pure CO_2_ Improved the Microbial Electrosynthesis of C4 and C6 Carboxylates. ACS Sustain. Chem. Eng..

[18] Zhou H (2021). Optimization of a newly developed electromethanogenesis for the highest record of methane production. J. Hazard. Mater..

[19] Rovira-Alsina L (2020). Thermophilic bio-electro CO_2_ recycling into organic compounds. Green. Chem..

[20] Yang HY (2021). Mixed-culture biocathodes for acetate production from CO_2_ reduction in the microbial electrosynthesis: Impact of temperature. Sci. Total Environ..

[21] Alqahtani MF (2019). Enrichment of Marinobacter sp. and Halophilic Homoacetogens at the Biocathode of Microbial Electrosynthesis System Inoculated With Red Sea Brine Pool. Front. Microbiol..

[22] Alqahtani MF (2021). Enrichment of salt-tolerant CO2–fixing communities in microbial electrosynthesis systems using porous ceramic hollow tube wrapped with carbon cloth as cathode and for CO2 supply. Sci. Total Environ..

[23] Siegert M, Li XF, Yates MD, Logan BE (2014). The presence of hydrogenotrophic methanogens in the inoculum improves methane gas production in microbial electrolysis cells. Front. Microbiol..

[24] Kobayashi H (2021). Analysis of a Methanogen and an Actinobacterium Dominating the Thermophilic Microbial Community of an Electromethanogenic Biocathode. Archaea.

[25] Izadi P, Gey MN, Schlüter N, Schröder U (2021). Bidirectional electroactive microbial biofilms and the role of biogenic sulfurin charge storage and release. iScience.

[26] Mateos R, Sotres A, Alonso RM, Moran A, Escapa A (2019). Enhanced CO2 Conversion to Acetate through Microbial Electrosynthesis (MES) by Continuous Headspace Gas Recirculation. Energies.

[27] Huang D, Song BY, Li MJ, Li XY (2018). Oxygen diffusion in cation-form Nafion membrane of microbial fuel cells. Electrochim. Acta.

[28] Trego AC (2021). First proof of concept for full-scale, direct, low-temperature anaerobic treatment of municipal wastewater. Bioresour. Technol..

[29] Galperin MY (2013). Genome Diversity of Spore-Forming. Firmicutes. Microbiol. Spectr..

[30] Aryal N, Tremblay PL, Lizak DM, Zhang T (2017). Performance of different Sporomusa species for the microbial electrosynthesis of acetate from carbon dioxide. Bioresour. Technol..

[31] Zeppilli M, Paiano P, Torres C, Pant D (2021). A critical evaluation of the pH split and associated effects in bioelectrochemical processes. Chem. Eng. J..

[32] Tremblay PL, Faraghiparapari N, Zhang T (2019). Accelerated h2 evolution during microbial electrosynthesis with sporomusa ovata. Catalysts.

[33] Philips J (2020). Extracellular electron uptake by acetogenic bacteria: Does H_2_ consumption favor the H_2_ evolution reaction on a cathode or metallic iron?. Front. Microbiol..

[34] Zhou J, Ning D (2017). Stochastic Community Assembly: Does It Matter in Microbial Ecology?. Microbiol. Mol. Biol. Rev..

[35] Chase JM (2010). Stochastic Community Assembly Causes Higher Biodiversity in More Productive Environments. Sci. (80-.)..

[36] Ning D (2020). A quantitative framework reveals ecological drivers of grassland microbial community assembly in response to warming. Nat. Commun..

[37] Nevin KP (2011). Electrosynthesis of organic compounds from carbon dioxide is catalyzed by a diversity of acetogenic microorganisms. Appl. Environ. Microbiol..

[38] Liu B, Kleinsteuber S, Centler F, Harms H, Sträuber H (2020). Competition Between Butyrate Fermenters and Chain-Elongating Bacteria Limits the Efficiency of Medium-Chain Carboxylate Production. Front. Microbiol..

[39] Thauer RK, Kaster AK, Seedorf H, Buckel W, Hedderich R (2008). Methanogenic archaea: Ecologically relevant differences in energy conservation. Nat. Rev. Microbiol..

[40] D’Amore R (2016). A comprehensive benchmarking study of protocols and sequencing platforms for 16S rRNA community profiling. BMC Genomics.

[41] Keating, C., Trego, A. C., Sloan, W., O\textquoterightFlaherty, V. & Ijaz, U. Z. Circular Economy of Anaerobic Biofilm Microbiomes: A Meta-Analysis Framework for Re-exploration of Amplicon Sequencing Data. *bioRxiv*10.1101/2020.12.23.424166 (2020).

[42] Thom C, Smith CJ, Moore G, Weir P, Ijaz UZ (2022). Microbiomes in drinking water treatment and distribution: A meta-analysis from source to tap. Water Res..

[43] Lahti, L., Shetty, S., Blake, T. & Salojarvi, J. Tools for microbiome analysis in R. Microbiome package. (2019).

[44] Foster ZSL, Sharpton TJ, Grünwald NJ (2017). Metacoder: An R package for visualization and manipulation of community taxonomic diversity data. PLOS Comput. Biol..

[45] Ning D, Deng Y, Tiedje JM, Zhou J (2019). A general framework for quantitatively assessing ecological stochasticity. Proc. Natl Acad. Sci..

[46] Verster AJ, Borenstein E (2018). Competitive lottery-based assembly of selected clades in the human gut microbiome. Microbiome.

[47] Trego AC (2021). Combined Stochastic and Deterministic Processes Drive Community Assembly of Anaerobic Microbiomes During Granule Flotation. Front. Microbiol..

