# Supplementary material for: A meta-analysis of acetogenic and methanogenic microbiomes in microbial electrosynthesis
Source: NPJ Biofilms Microbiomes. 2022 Sep 23;8:73. doi: 10.1038/s41522-022-00337-5 (PMC9500080; doi:10.1038/s41522-022-00337-5)
Supplement: Supplementary file 1 — Supplementary Material [file 41522_2022_337_MOESM1_ESM.pdf]

A meta-analysis of cathodic and planktonic microbiomes in microbial electrosynthesis

Simon Mills[a,#], Paolo Dessì[b,#], Deepak Pant[c], Pau Farràs[b], William T. Sloan[d], Gavin Collins[a,], Umer Z. Ijaz[d]*

[a] *Microbiology, School of Natural Sciences and Ryan Institute, National University of Ireland Galway, University Road, Galway, H91 TK33, Ireland*

[b] *Energy Research Centre, Ryan Institute, National University of Ireland Galway, University Road, H91 TK33 Galway, Ireland*

[c] *Separation and Conversion Technology, Flemish Institute for Technological Research (VITO), Mol, Belgium*

[d] *Infrastructure and Environment Research Division, School of Engineering, University of Glasgow, Oakfield Avenue, Glasgow G12 8LT, United Kingdom*

Supplementary information

[#] **These authors contributed equally to the manuscript.**

***** Corresponding author: Dr Gavin Collins, National University of Ireland Galway, University Road, Galway, H91 TK33, Ireland, e-mail: gavin.collins@nuigalway.ie

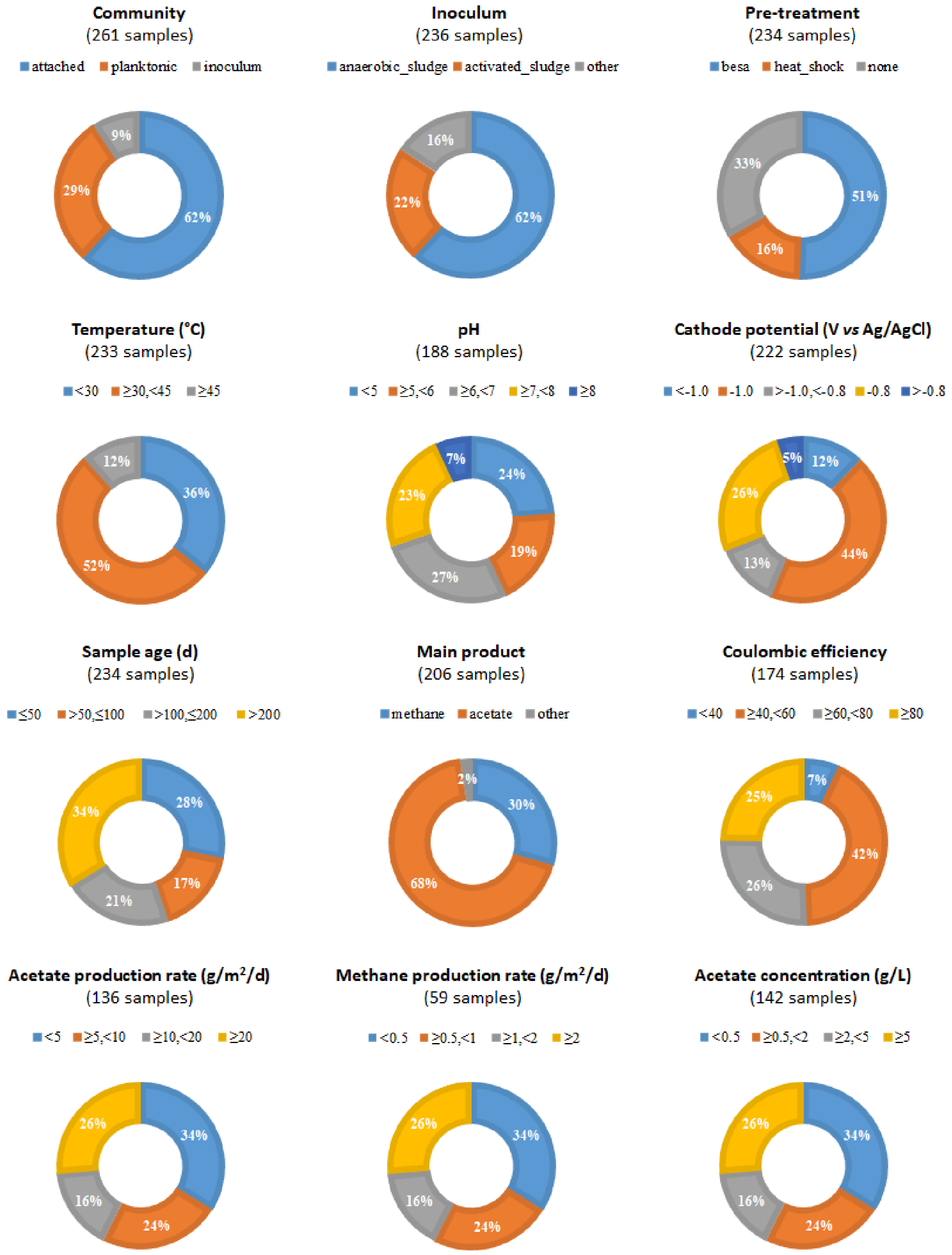

Supplementary Figure 1. Sample metadata distributions. Overview of the proportion of samples which were assigned to each variable investigated in the study.

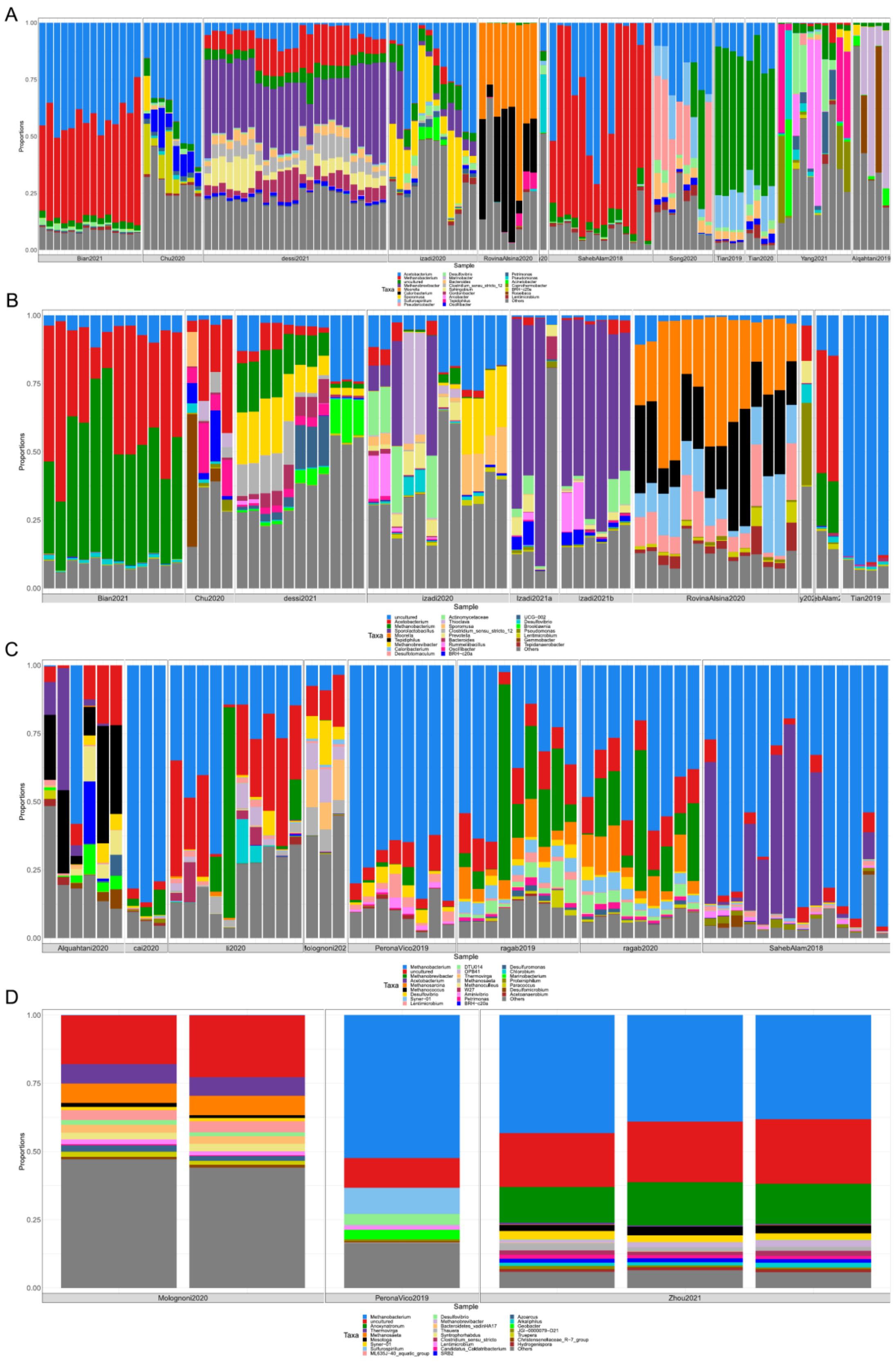

Supplementary Figure 2. Most abundant taxa in acetogenic and methanogenic cells.
Taxa bars depicting the 25 most abundant Genera in (A) cathodic acetogenic communities

(B) planktonic acetogenic communities (C) cathodic attached communities (D) and planktonic methanogenic communities, grouped by study. Others represents taxa outside of the top 25

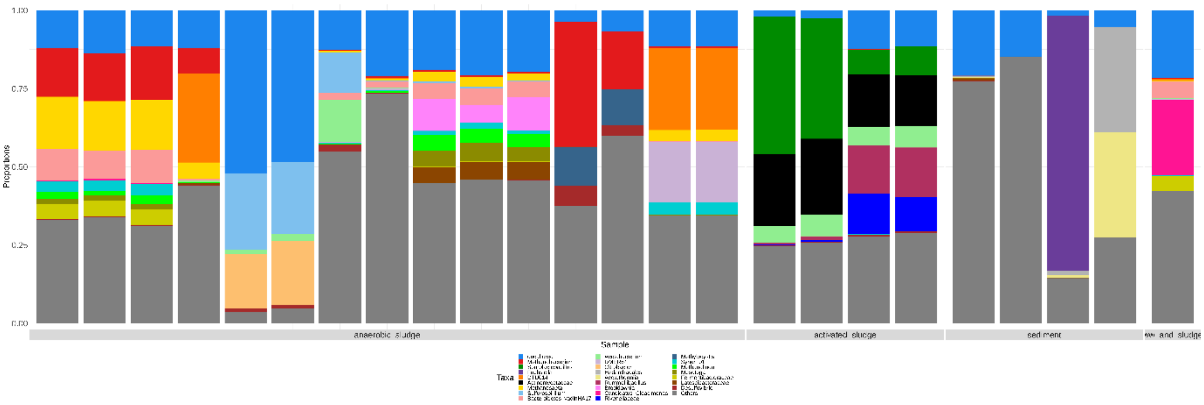

Supplementary Figure 3. Most abundant tax in inoculum. Taxa bars depicting the 25 most abundant Genera in inoculum communities, grouped by inoculum type. Others represents taxa outside of the top 25

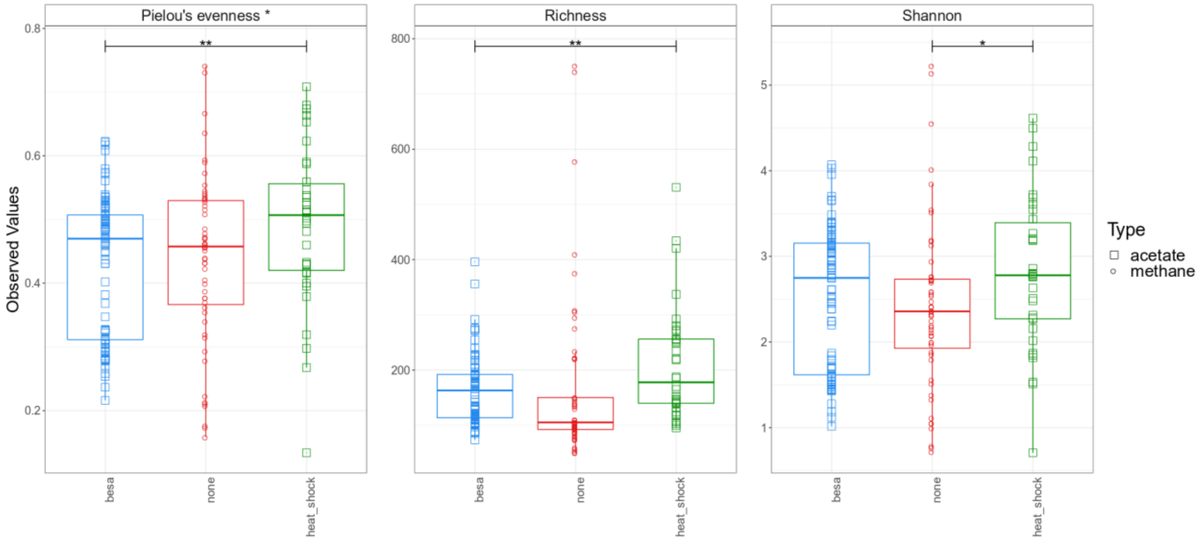

Supplementary Figure 4. Alpha Diversity Indices; Pielou's Evenness, Rarefied Richness and Shannon Entropy (Lines of significance depict significant differences as follows: * ($p < 0.05$), ** ($p < 0.01$), or *** ($p < 0.001$) based on ANOVA) for samples from acetogenic and methanogenic MES cells, where samples are grouped by pre-treatment type.

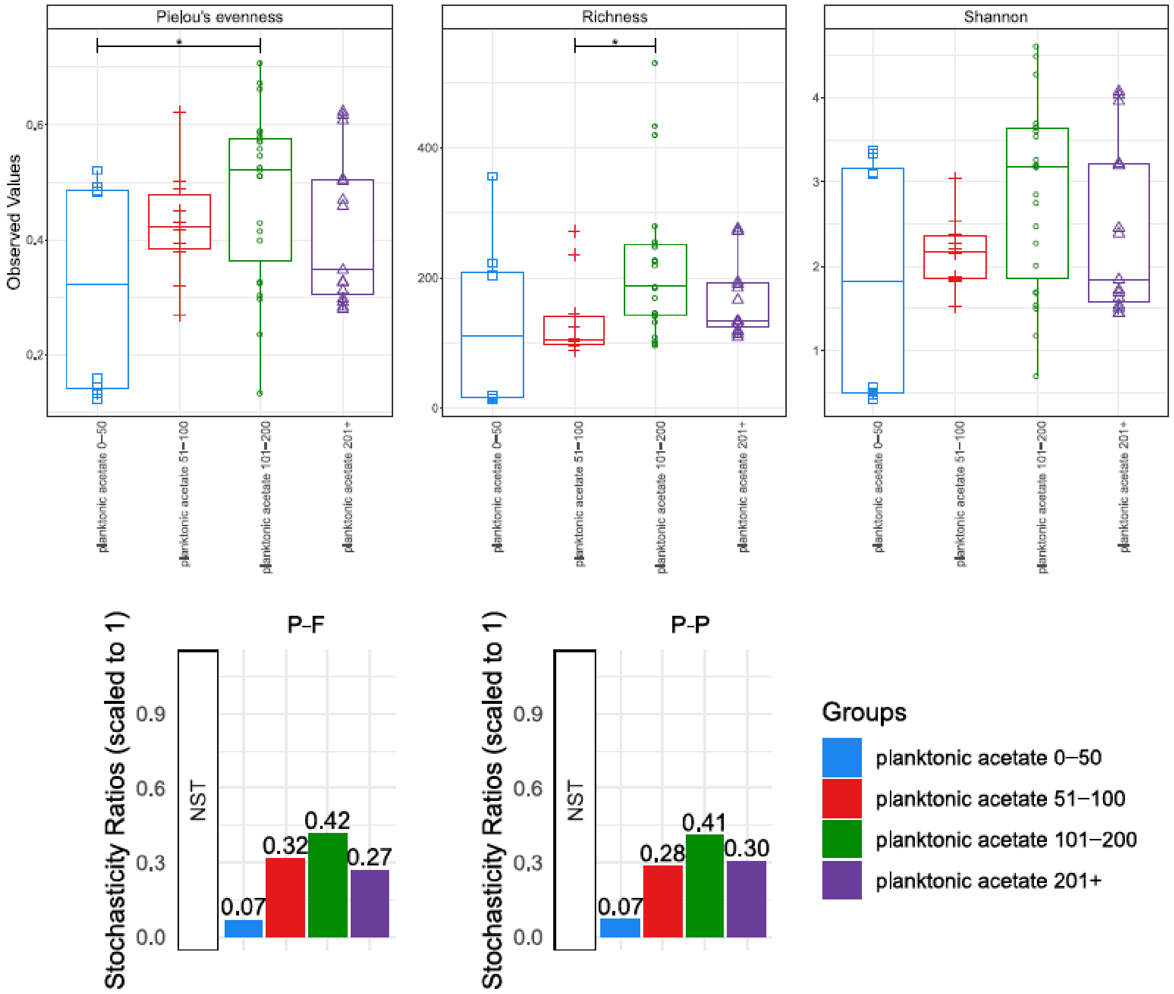

Supplementary Figure 5. (A) Alpha Diversity Indices, Pielou's Evenness, Rarefied Richness and Shannon Entropy for planktonic acetogenic communities, grouped by the number of days of cell operation at which the sample was taken. In the boxplots, center value lines indicate the median, boxes indicate the lower/upper quartiles (25%/75%) and lines extending parallel from the boxes (whiskers) show the variability outside the upper and lower quartiles. (B) Normalized stochasticity ratio (NST) using Ružička metric and Taxa-Richness constraints of proportional-fixed (P-F) and proportional-proportional (P-P) which stipulates that the probabilities of taxa occurrence are proportional to the observed occurrence frequencies, and taxon richness in each sample is either fixed or proportional.

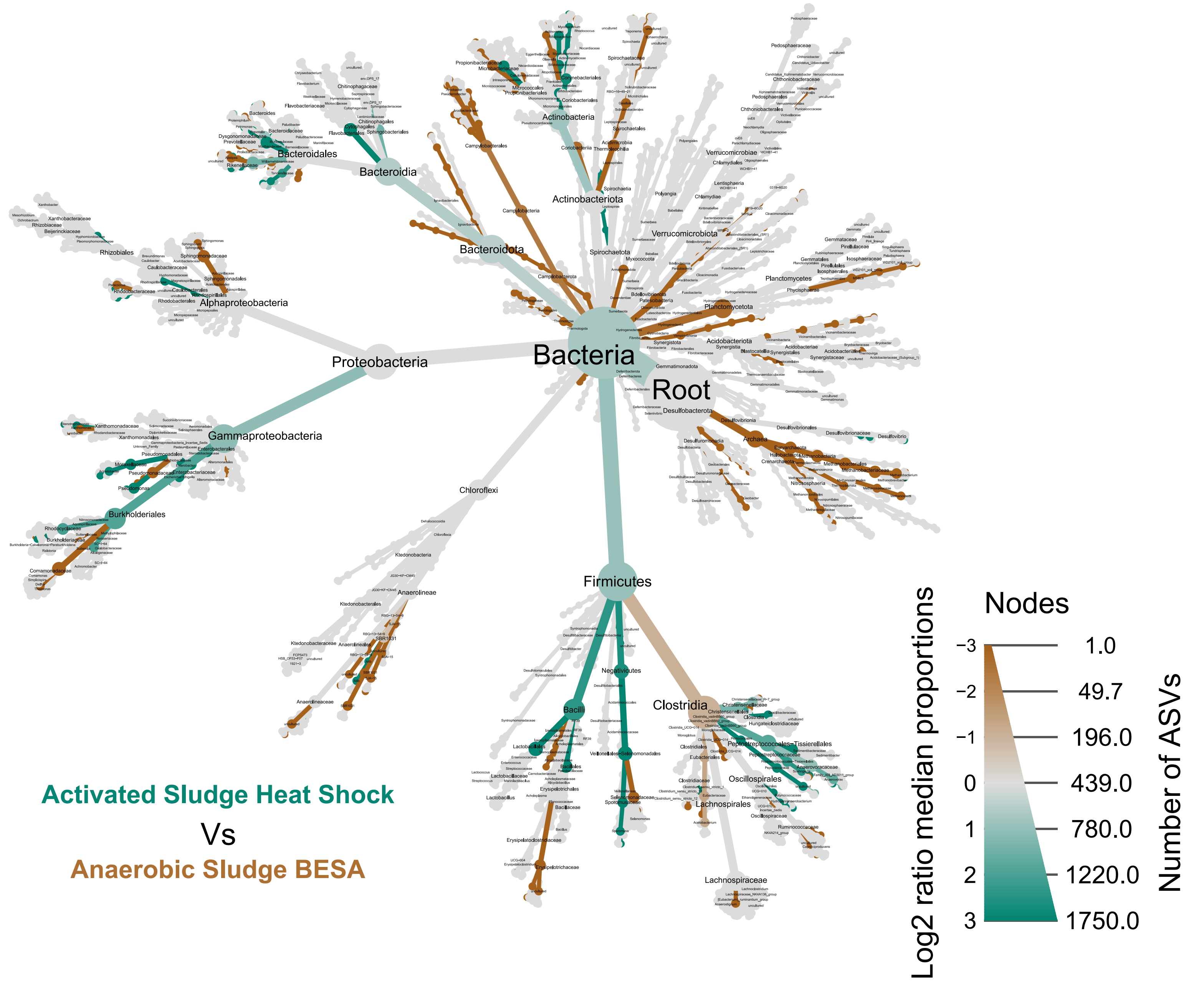

Supplementary Figure 6. Full sized version of heat tree depicted in Figure 4 of the main text. Differential heat tree differently abundant taxa between two groups based on seeding strategy (heat shocked activated sludge Vs BESA treated anaerobic sludge). The circle size and the colour intensity reflect the species abundance and the log2 median proportion between the two groups respectively.

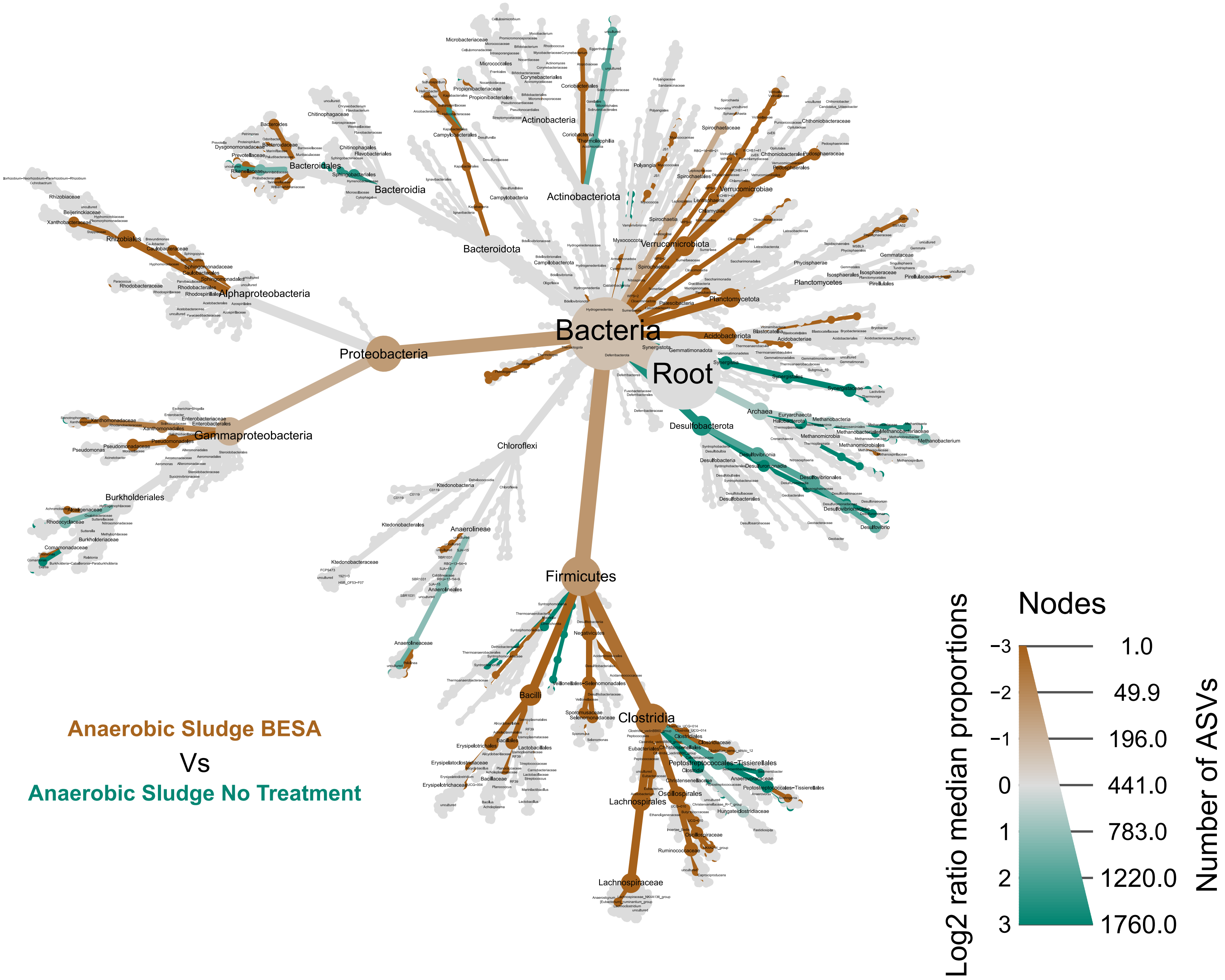

Supplementary Figure 7. Full sized version of heat tree depicted in Figure 4 of the main text. Differential heat tree dep differently abundant taxa between two groups based on seeding strategy (untreated anaerobic sludge Vs BESA treated anaerobic sludge). The circle size and the colour intensity reflect the species abundance and the log2 median proportion between the two groups respectively.

Methodology Justification

The 25 most abundant genera in several larger studies from (A) individual biom files and (B) when they are part of the collated biom files are provided below to offer visual cues for assessing similarities. Additionally, for each of these studies we have performed a Mantel test by considering all the ASVs we obtained for individual studies and the ASVs we were able to resolve at sequence level (which could be found in the reference database). The Mantel test was calculated using Bray-Curtis distance to find the similarity between the same samples. The correlation values along with the significances are provided below.

(A) Individual biom file

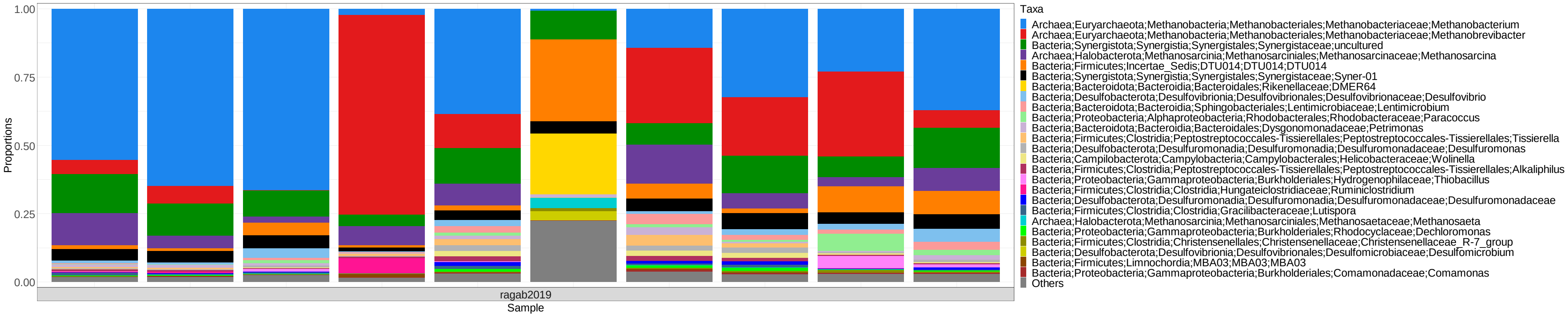

(B) Collated biom file

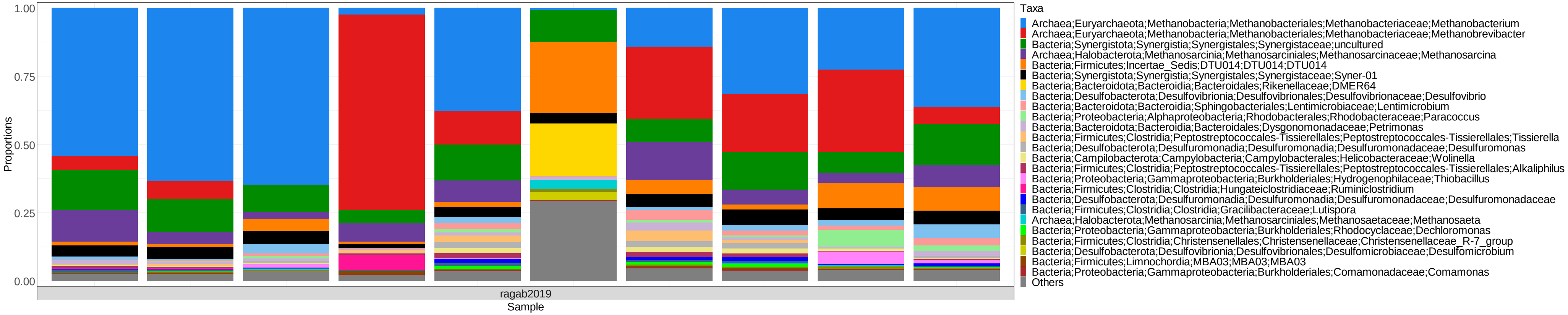

Supplementary Figure 8. Ragab et al 2019 (Mantel R = 0.997 p < 0.001 ***)

(A) Individual biom file

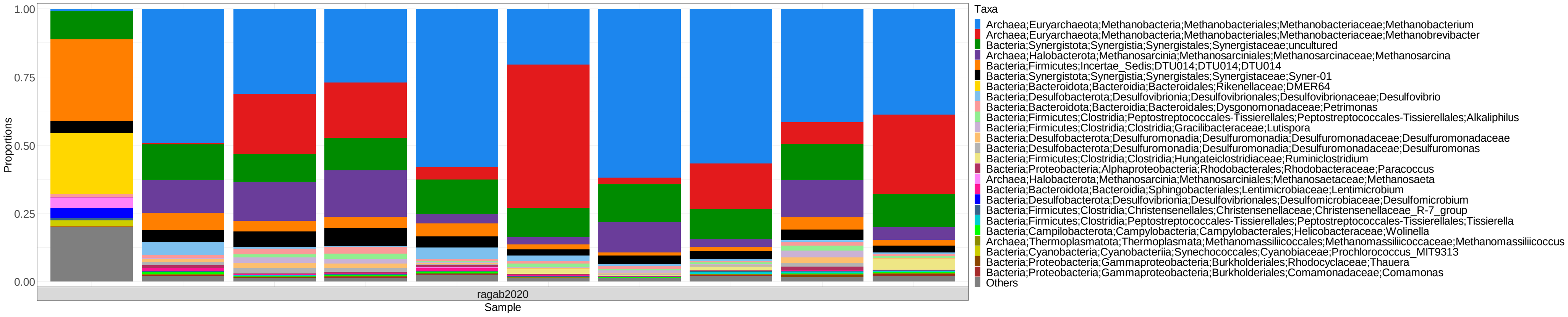

(B) Collated biom file

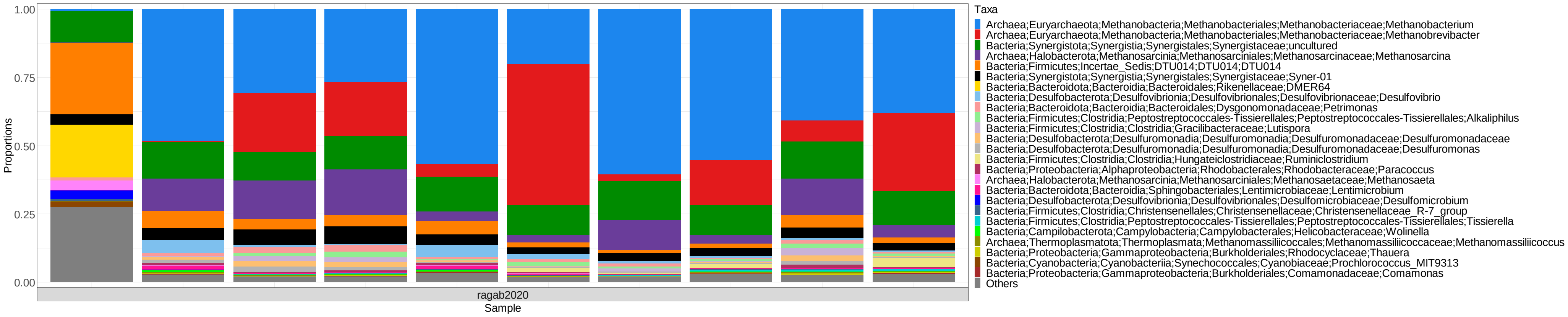

Supplementary Figure 9. Ragab et al 2020 (Mantel R = 0.993 $p < 0.001$ ***)

(A) Individual biom file

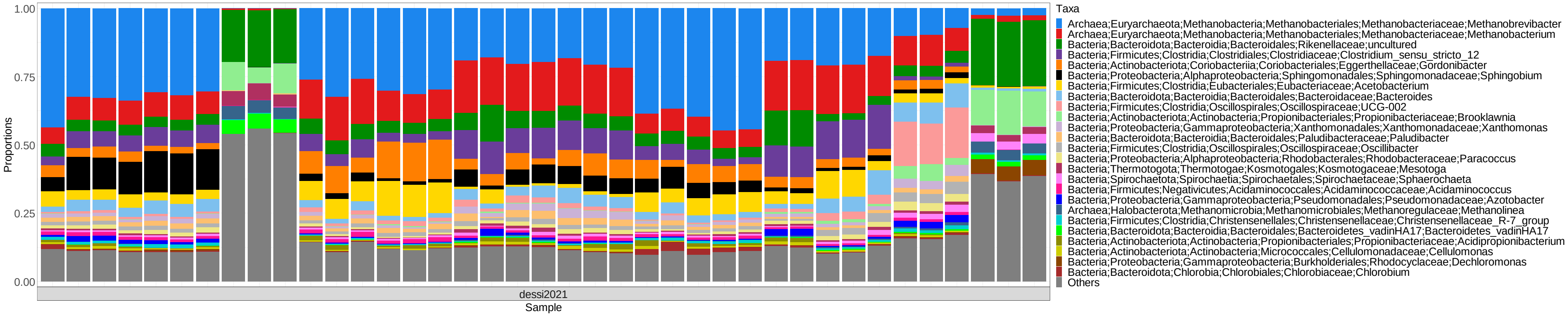

(B) Collated biom file

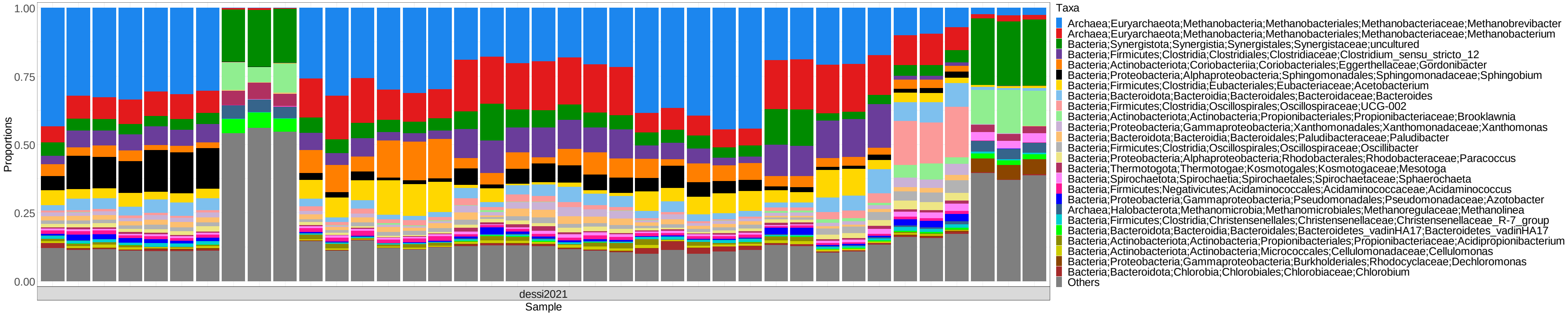

Supplementary Figure 10. Dessi et al 2021 (Mantel R = 0.998 p < 0.001 ***)

(A) Individual biom file

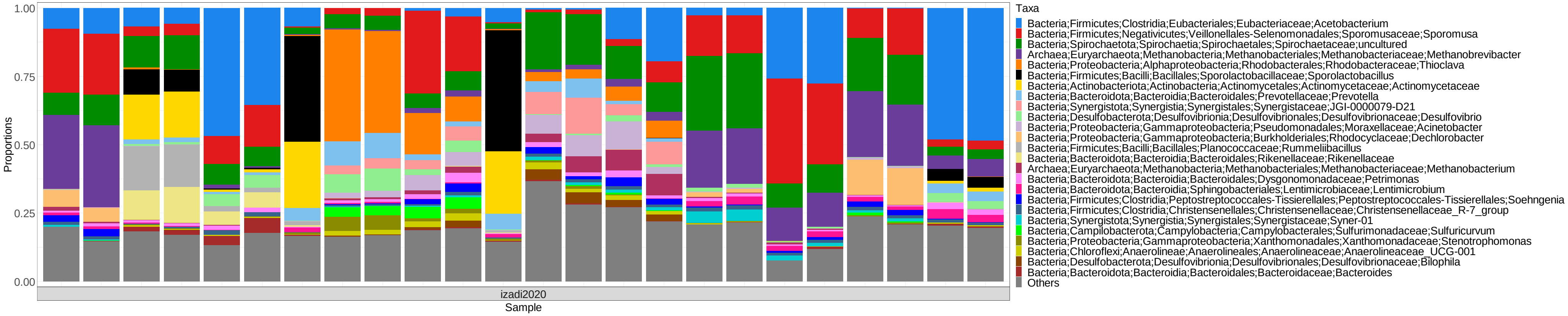

(B) Collated biom file

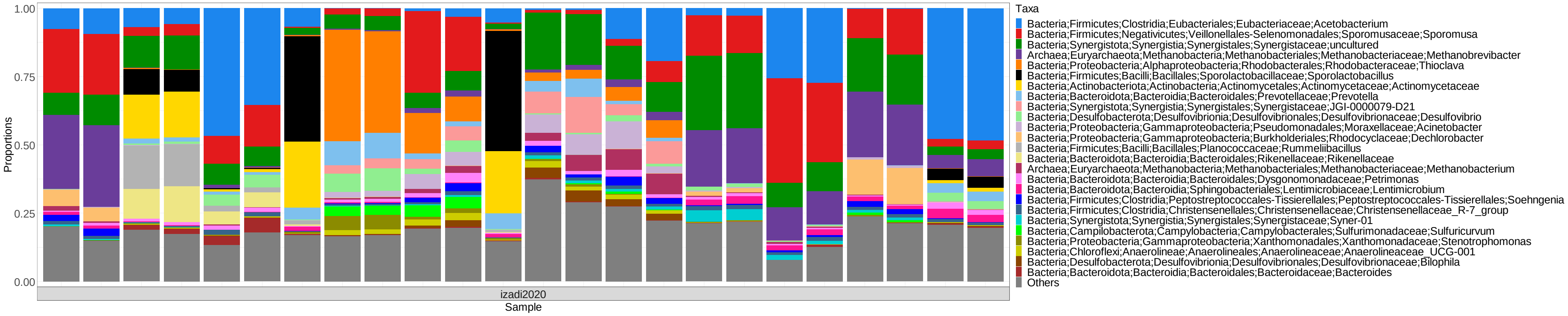

Supplementary Figure 11. Izadi et al 2020 (Mantel R = 0.996 p < 0.001 ***)

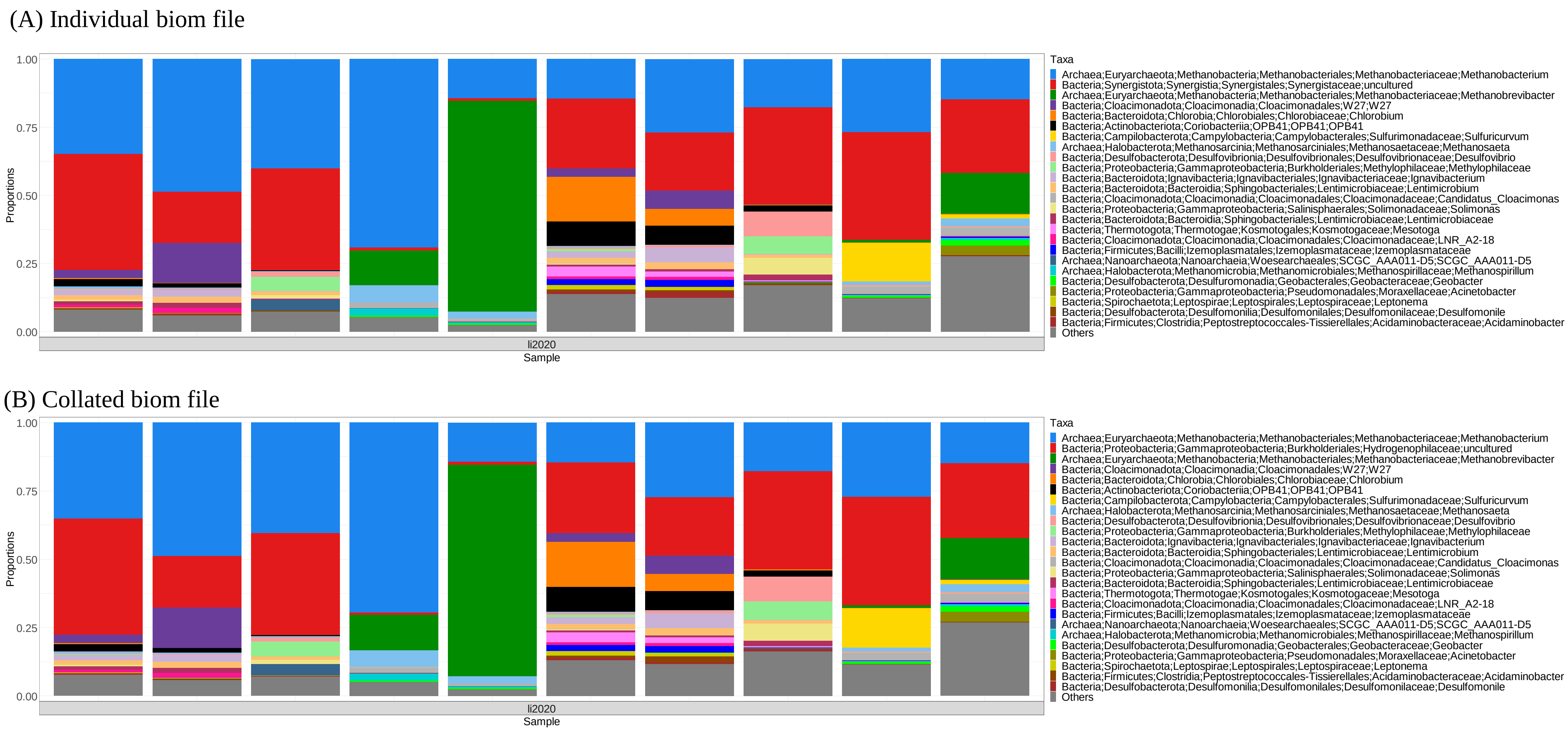

Supplementary Figure 12. Li et al 2020 (Mantel R = 0.997 p < 0.001 ***)

(A) Individual biom file

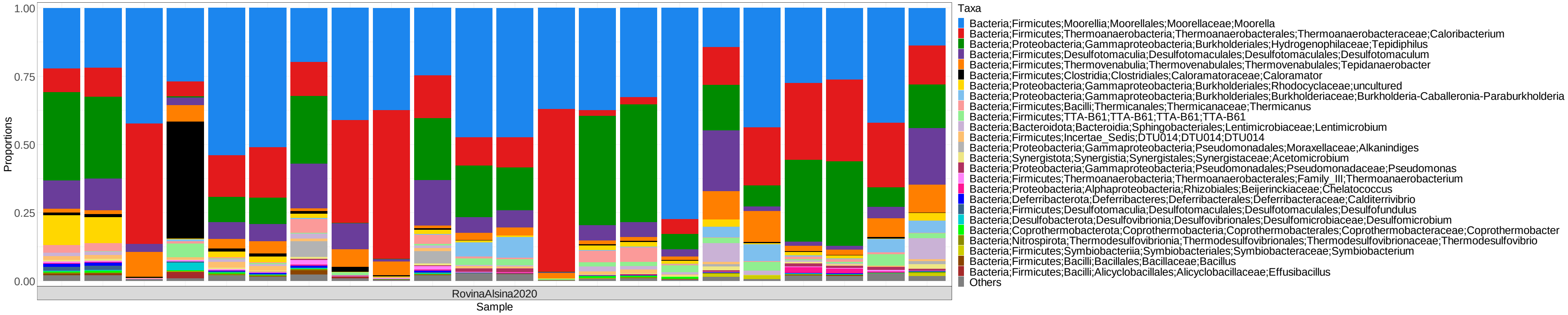

(B) Collated biom file

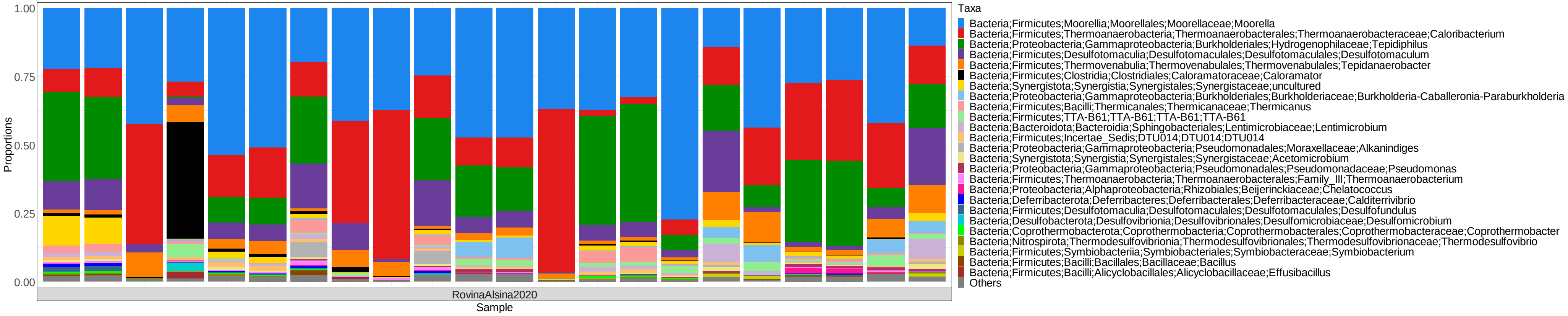

Supplementary Figure 13. Rovina Alsina et al 2020 (Mantel R = 0.983 $p < 0.001$ ***)

(A) Individual biom file

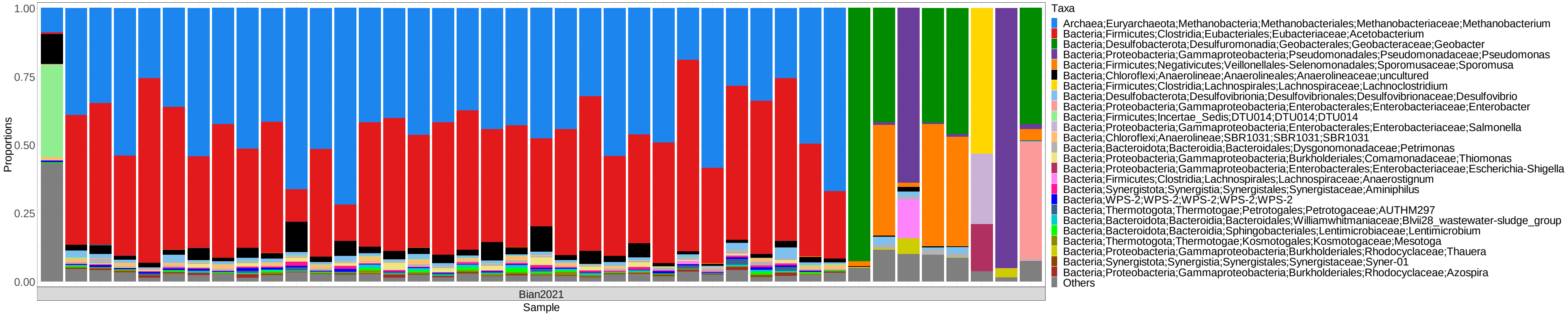

(B) Collated biom file

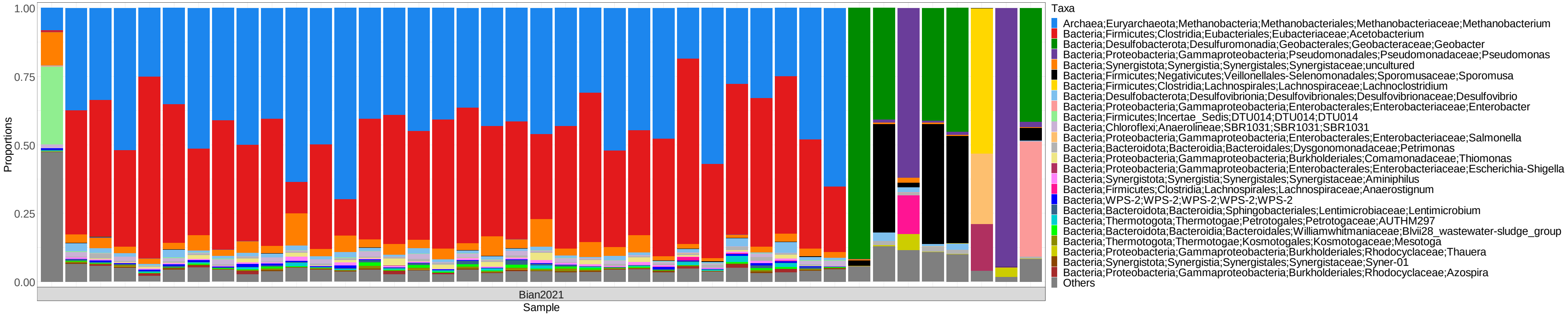

Supplementary Figure 14. Bian et al 2021 (Mantel R = 0.998 $p < 0.001$ ***)

(A) Individual biom file

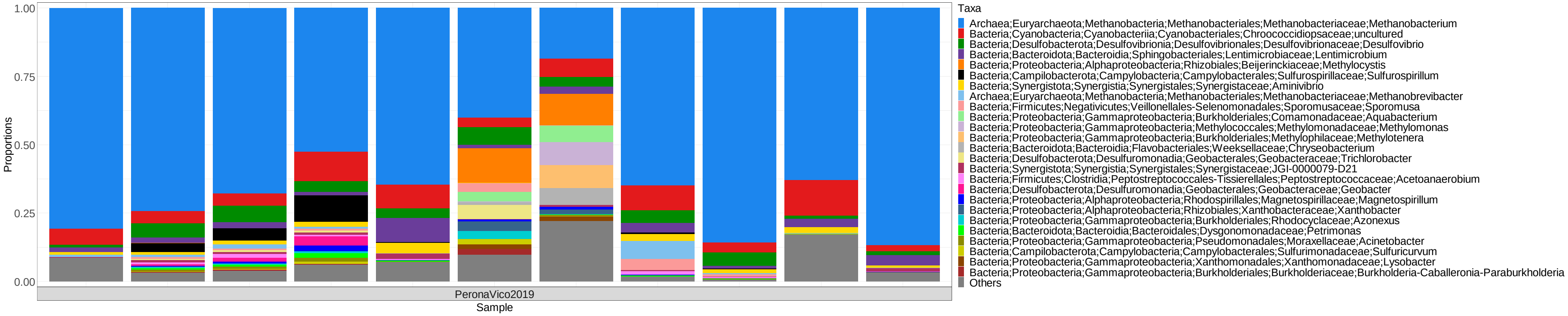

(B) Collated biom file

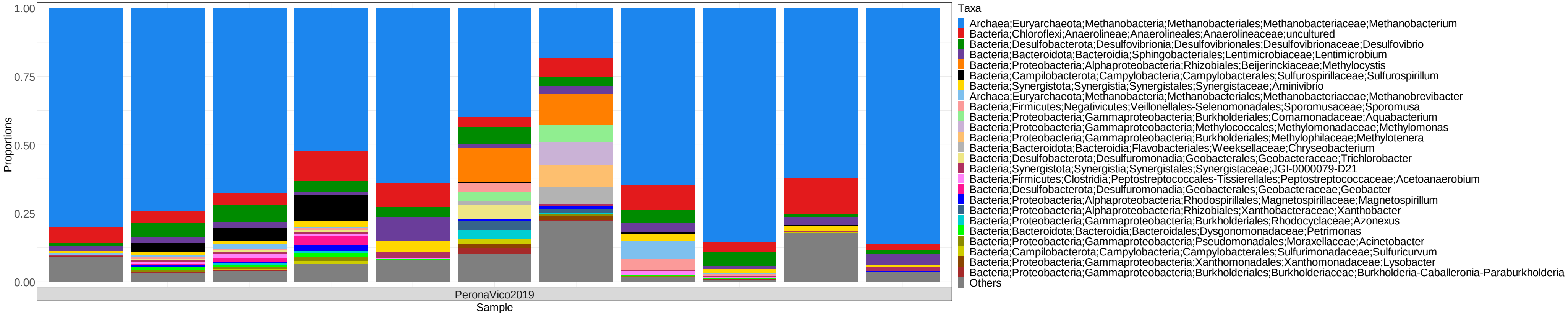

Supplementary l Figure 15. Perona-Vico et al 2019 (Mantel R = 0.999 p < 0.001 ***)

Supplementary Table 1. List of research articles included in this meta-analysis

Authors	Title	Year	Journal	DOI	Bioproject number
Alqahtani et al.	Enrichment of *Marinobacter* sp. and halophilic homoacetogens at the biocathode of microbial electrosynthesis system inoculated with red sea brine pool	2019	Frontiers in microbiology	10.3389/fmicb.2019.02563	PRJNA545216
Alqahtani et al.	Enrichment of salt-tolerant CO_2–fixing communities in microbial electrosynthesis systems using porous ceramic hollow tube wrapped with carbon cloth as cathode and for CO_2 supply	2020	Science of the total environment	10.1016/j.scitotenv.2020.142668	PRJNA631224
Bian et al.	Resistance assessment of microbial electrosynthesis for biochemical production to changes in delivery methods and CO_2 flow rates	2021	Bioresource technology	10.1016/j.biortech.2020.124177	Data provided by Authors
Cai et al.	Semiquantitative detection of hydrogen-associated or hydrogen-free electron transfer within methanogenic biofilm of microbial electrosynthesis	2020	Applied and environmental microbiology	10.1128/AEM.01056-20	PRJNA629292
Chu et al.	Waste C1 gases as alternatives to pure CO_2 improved the microbial electrosynthesis of C4 and C6 carboxylates	2020	ACS sustainable chemistry & engineering	10.1021/acssuschemeng.0c02515	Data provided by Authors
Dessì et al.	Carboxylic acids production and electrosynthetic microbial community evolution under different CO_2 feeding regimens	2021	Bioelectrochemistry	10.1016/j.bioelechem.2020.107686	PRJNA659986
Izadi et al.	Parameters influencing the development of highly conductive and efficient biofilm during microbial electrosynthesis: the importance of applied potential and inorganic carbon source	2020	NPJ biofilms and microbiomes	10.1038/s41522-020-00151-x	PRJNA663785
Izadi et al.	Enhanced bio-production from CO_2 by microbial electrosynthesis (MES) with continuous operational mode	2021	Faraday discussion	10.1039/d0fd00132e	Data provided by Authors
Izadi et al.	The effect of the polarised cathode, formate and ethanol on chain elongation of acetate in microbial electrosynthesis	2021	Applied energy	10.1016/j.apenergy.2020.116310	Data provided by Authors
Li et al.	Startup cathode potentials determine electron transfer behaviours of biocathodes catalysing CO_2 reduction to CH_4 in microbial electrosynthesis	2020	Journal of CO_2 utilization	10.1016/j.jcou.2019.09.013	PRJDB8094
Molognoni et al.	How operational parameters affect electromethanogenesis in a bioelectrochemical power-to-gas prototype	2020	Frontiers in energy research	10.3389/fenrg.2020.00174	PRJNA627951

Perona-Vico et al.	[NiFe]-hydrogenases are constitutively expressed in an enriched *Methanobacterium* sp. population during electromethanogenesis	2019	Plos one	10.1371/journal.pone.0215029	PRJNA481232
Ragab et al.	Evidence of spatial homogeneity in an electromethanogenic cathodic microbial community	2019	Frontiers in microbiology	10.3389/fmicb.2019.01747	PRJNA541055
Ragab et al.	Effects of set cathode potentials on microbial electrosynthesis system performance and biocathode methanogen function at a metatranscriptional level	2020	Scientific reports	10.1038/s41598-020-76229-5	PRJNA543631
Rovira-Alsina et al.	Thermophilic bio-electro CO_2 recycling into organic compounds	2020	Green chemistry	10.1039/d0gc00320d	PRJNA557160
Roy et al.	Direct utilization of industrial carbon dioxide with low impurities for acetate production via microbial electrosynthesis	2021	Bioresource technology	10.1016/j.biortech.2020.124289	PRJNA659908
Saheb-Alam et al.	Effect of start-up strategies and electrode materials on carbon dioxide reduction on biocathodes	2018	Applied and environmental microbiology	10.1128/AEM.02242-17	PRJNA412029
Song et al.	Hydrothermal synthesis of MoS_2 nanoflowers for an efficient microbial electrosynthesis of acetate from CO_2	2020	Journal of CO_2 utilization	10.1016/j.jcou.2020.101231	Data provided by Authors
Tian et al.	Mo_2C-induced hydrogen production enhances microbial electrosynthesis of acetate from CO_2 reduction	2019	Biotechnology for biofuels	10.1186/s13068-019-1413-z	Data provided by Authors
Tian et al.	Artificial electron mediator with nanocubic architecture highly promotes microbial electrosynthesis from carbon dioxide	2020	ACS sustainable chemistry & engineering	10.1021/acssuschemeng.0c01276	Data provided by Authors
Yang et al.	Mixed-culture biocathodes for acetate production from CO_2 reduction in the microbial electrosynthesis: Impact of temperature	2021	Science of the total environment	10.1016/j.scitotenv.2021.148128	Data provided by Authors
Zhou et al.	Optimization of a newly developed electromethanogenesis for the highest record of methane production	2021	Journal of hazardous materials	10.1016/j.jhazmat.2020.124363	Data provided by Authors
